# *Atri-U*: assisted image analysis in routine cardiovascular magnetic resonance volumetry of the left atrium

**DOI:** 10.1186/s12968-021-00791-8

**Published:** 2021-11-11

**Authors:** Constantin Anastasopoulos, Shan Yang, Maurice Pradella, Tugba Akinci D’Antonoli, Sven Knecht, Joshy Cyriac, Marco Reisert, Elias Kellner, Rita Achermann, Philip Haaf, Bram Stieltjes, Alexander W. Sauter, Jens Bremerich, Gregor Sommer, Ahmed Abdulkadir

**Affiliations:** 1 Department of Radiology, University Hospital Basel, University of Basel, Basel, Switzerland; 2 Department of Research and Analysis, University Hospital Basel, University of Basel, Basel, Switzerland; 3 Department of Radiology, University Children’s Hospital Basel, University of Basel, Basel, Switzerland; 4 Department of Cardiology, University Hospital Basel, University of Basel, Basel, Switzerland; 5 Medical Physics, Department of Radiology, University Medical Center Freiburg, Freiburg, Germany; 6 University Hospital of Old Age Psychiatry and Psychotherapy, University of Bern, Bern, Switzerland; 7 Center for Biomedical Image Computing and Analytics, Perelman School of Medicine, University of Pennsylvania, Philadelphia, PA USA

**Keywords:** Magnetic resonance imaging, Heart atria, Artificial intelligence, Workflow, Atrial fibrillation, Biplane area-length method

## Abstract

**Background:**

Artificial intelligence can assist in cardiac image interpretation. Here, we achieved a substantial reduction in time required to read a cardiovascular magnetic resonance (CMR) study to estimate left atrial volume without compromising accuracy or reliability. Rather than deploying a fully automatic black-box, we propose to incorporate the automated LA volumetry into a human-centric interactive image-analysis process.

**Methods and results:**

*Atri-U*, an automated data analysis pipeline for long-axis cardiac cine images, computes the atrial volume by: (i) detecting the end-systolic frame, (ii) outlining the endocardial borders of the LA, (iii) localizing the mitral annular hinge points and constructing the longitudinal atrial diameters, equivalent to the usual workup done by clinicians. In every step human interaction is possible, such that the results provided by the algorithm can be accepted, corrected, or re-done from scratch. *Atri-U* was trained and evaluated retrospectively on a sample of 300 patients and then applied to a consecutive clinical sample of 150 patients with various heart conditions. The agreement of the indexed LA volume between *Atri-U* and two experts was similar to the inter-rater agreement between clinicians (average overestimation of 0.8 mL/m^2^ with upper and lower limits of agreement of − 7.5 and 5.8 mL/m^2^, respectively). An expert cardiologist blinded to the origin of the annotations rated the outputs produced by *Atri-U* as acceptable in 97% of cases for step (i), 94% for step (ii) and 95% for step (iii), which was slightly lower than the acceptance rate of the outputs produced by a human expert radiologist in the same cases (92%, 100% and 100%, respectively). The assistance of *Atri-U* lead to an expected reduction in reading time of 66%—from 105 to 34 s, in our in-house clinical setting.

**Conclusions:**

Our proposal enables automated calculation of the maximum LA volume approaching human accuracy and precision. The optional user interaction is possible at each processing step. As such, the assisted process sped up the routine CMR workflow by providing accurate, precise, and validated measurement results.

**Supplementary Information:**

The online version contains supplementary material available at 10.1186/s12968-021-00791-8.

## Background

Left atrial (LA) enlargement is frequently associated with atrial fibrillation, thromboembolic events and eventually death [1, 2]. Even in the absence of atrial fibrillation, the abnormal shape or size of the LA has been linked to stroke, heart failure and major adverse cardiac events [3].

Cardiovascular magnetic resonance (CMR) is the reference imaging modality for measuring cardiac chamber volumes, including the LA [4]. LA dilatation is best recognized by measuring the maximum LA volume (${LAV}_{max}$) at ventricular end-systole.^1^ It is usually measured using two long-axis (LAx) multiphase cine images, the two chamber- and four chamber- (2ch- and 4ch-, respectively) views. This analysis is known as the biplane area‐length method and has been validated for routine CMR assessment both in sinus rhythm and in atrial fibrillation [5]. It comprises the following steps: (i) identifying the frame of end-systolic (ES) phase, (ii) outlining the LA in the two views and (iii) drawing longitudinal atrial diameters. When using dedicated software, a human expert performs these steps manually in a few minutes. However, this time adds to the total time required for the evaluation and reporting of a whole-heart CMR study, which typically takes 30 min or more. An alternative method of calculating the LA volume from multislice cine stacks offers additional options for quantification of phasic function and strain but is even more time consuming [6].

We aimed at reducing the time required to compute ${LAV}_{max}$ without loss of reliability, by assisting the human expert in the CMR assessment. For this purpose, we developed *Atri-U,* an image analysis pipeline that reproduces above-mentioned steps (i)–(iii) of the biplane area-length method. *Atri-U* was then integrated into an established clinical workup that provides the human expert a way to review and revise the outputs in a familiar interface. As final outcomes, we evaluate the frequency and severity of disagreements between *Atri-U* and an expert and the resulting gain in time by checking, accepting, and eventually processing only cases that are rated with insufficient quality.

## Methods

### Datasets

LAx cines from 1697 patients, admitted to our institution between June 2010 and June 2019 for whole-heart clinical routine CMR imaging, formed the initial sample of the study. Exclusion criteria and age limits (≥ 18 years) were applied and 1379 cases remained, from which $N=300$ pairs of 2ch- and 4ch-view cines were selected for the training and evaluation of *Atri-U* (samples A, B and C for training, validation and testing, respectively, see Table 1). The exclusion criteria, the selection process (adapted from [7]) and acquisition parameters are listed in Additional file 1: Table S1 and Figure S1. Additionally, $N=150$ consecutively acquired CMR studies starting July 2019 were used for assessment of the time saving achieved with *Atri-U* (samples D_1_/D_2_). The reference standard was created, as previously described [8], by a radiologist (TAD) and a radiologist in training (CA) for samples A, B and C and by a senior radiologist subspecialized in cardiovascular imaging with 6 years of experience (GS) for sample D_1_ (for details see Additional file 1: Section E2). Demographic and clinical characteristics were extracted from radiological reports and are summarized in Additional file 1: Table S2 and Figure S2. Finally, in order to extend the field of application to 3D data of the LA, multislice axial stacks of CMR cines were also analyzed ($N=65$). Full details of the 3D segmentation task, as well as an intra-subject correlation of 2D and 3D volumetry, are provided in the Additional file 1: Section E3). The study was approved by the *Ethikkommission Nordwest- und Zentralschweiz* ethics committee (project-ID 2019-01637).

**Table 1:** Characteristics and use of data samples in chronological order

	Training and evaluation of *Atri-U*			Evaluation in clinical setting		Evaluation aspect
Sample	A	B	C	D_1_	D_2_	
Ground truth of segmentation, landmarks and ES	TA and CA (100 each)	TA and CA	TA and CA (25 each)	GS	n.a	*Atri-U* modules
Training of *Atri-U*	✓	X	X	X	X	
Evaluation of modules	X	✓	✓	X	X	
Evaluation of $LA{V}_{\mathrm{max}}$	X	✓	✓	✓	X	Atrial volume
Proposal of segmentation, landmarks and ES	n.a	n.a	n.a	GS and *Atri-U*	*Atri-U*	Clinical value (time and quality)
Independent check	n.a	n.a	n.a	PH	PH	
Count	200	50	50	50	100	
Sample type	1/3 random subsample, 2/3 selected subsample		Random sample^#^	Consecutive sample		
Acquisition interval	2014–06/2018		07/2018–06/2019	After 07/2019		
Magnetic field strength (1.5/3 Tesla)	150/100		25/25	112/38		
Atrial dilatation	64 (26%)		16 (32%)	30 (20%)		
Structural heart disease*	189 (76%)		35 (70%)	103 (69%)		

The convolutional neural networks were trained on sample A, validated on sample B and *Atri-U* was finally tested on sample C. The time saving value was elaborated on the partially overlapping samples D_1_ and D_2_, processed by a senior radiologist and *Atri-U*, respectively, and rated by a senior cardiologist. *Details on the subtypes of structural heart disease are listed in Additional file 1: Table S2 and Figure S2. ^#^While maintaining the ratio of magnetic field strength at 1:1

*ES* = end-systole, CA, GS, PH, TA = authors initials, ${LAV}_{max}$ = maximum left atrial volume, n.a. = not applicable, ✓ (tick) means available/completed, X means not available/not performed

### Evaluation of *Atri-U* in clinical setting

In its clinical application, *Atri-U* generates proposals for all steps of the biplane area-length method, as described in “*Atri-U *modules that implement the biplane area-length method” section, which are then made available to the human expert for review and optional correction. The added value of *Atri-U* was measured by the average time saving per case. For this evaluation, *Atri-U* processed $N=150$ consecutively acquired cases (sample D_2_), the chronologically first $N=50$ of which had been also processed by a senior radiologist (GS, sample D_1_), resulting in $N=200$ annotations (see Table 1). A cardiologist with 7 years of experience in cardiac imaging (PH), who was not involved in the creation of the reference standard and was not told that 50 of the LAx cine pairs appeared twice, scored all annotations in randomized order. He used a categorical rating, scoring the quality of each module separately (ES frame detection; segmentation of LA; diameter placement). The given score was based on the estimated time required to obtain an acceptable result as follows: Score 0: 100 percent time saving (no correction needed); Score 1: 50 percent time saving (minor correction needed); Score 2: no time saving (major correction needed). Expected time saving was computed with the assumption that the better the proposed annotation by *Atri-U* was, the more time could be saved. The total expected time saved minus the actual time required for the scoring yielded an estimate of the time saving for a given cine pair. Additionally, the ${LAVI}_{max}$ was compared in the subset of 50 cases that appeared in both samples D_1_ and D_2_ (processed by the radiologist and *Atri-U*, respectively), focusing on the correlation and the difference in cases that did receive a score other than 2.

### *Atri-U* modules that implement the biplane area-length method

#### Overview

*Atri-U* was implemented with fully convolutional neural networks followed by conventional morphometric and geometrical computations to mimic the steps performed by humans as detailed in the following paragraphs. In the scope of the proof of concept, we kept a single model.

#### Module 1 that implements detection of ES frame

The mitral valve states were classified on each 4Ch-view frame using a fine-tuned fully convolutional neural network [9] with the closed states as the positive and the open states as the negative class. The number of channels in the four convolutional layers were 64, 128, 256, and 512, respectively. The binary classification was optimized via stochastic gradient decent with momentum using cross-entropy loss with closed states as the positive and the open states as the negative label (for more details on the network architectures see Additional file 1: Section E2). Upon prediction, the last frame of the largest block of closed states was defined as the ES frame and the performance metrics of accuracy, recall and precision were computed for each CMR study.

#### Module 2 that implements segmentation of left atrium

Pixel-wise automated segmentation of the LA in both the 2Ch- and 4Ch-view was performed with the U-Net [10, 11]. The overlap of two LA segmentations was quantitatively evaluated using established performance metrics (for details see Additional file 1: Table S3 and [12]).

#### Module 3 that implements placement of longitudinal atrial diameters

**Fig. 1:**
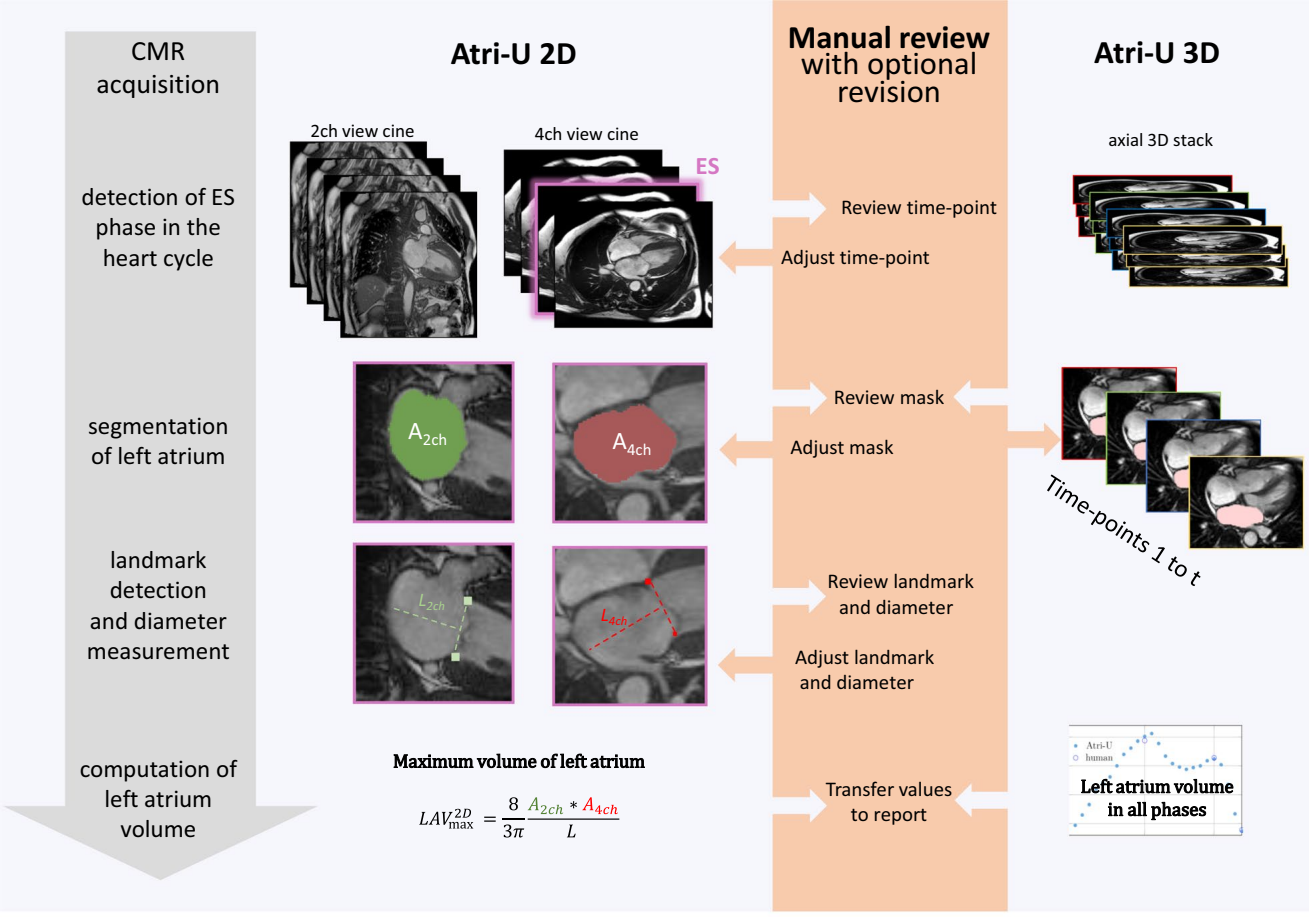
Automated calculation of the left atrial (LA) volume from long-axis CMR cines and 3D cines with *Atri-U*. The existing workflow enables the manual review of automated predictions at each individual step of the biplane area-length method (orange column). If revision is required the corrected predictions are used to recalculate maximum LA volume (LAV_max_, where A_2ch_ and A_4ch_ are LA areas and L is the minimum of two longitudinal diameters). For the 3D cines, the steps of frame detection and landmark localization do not apply as the volume is calculated from the sum of atrial area on each slice times the slice thickness (see Additional file 1: Section E3). *ES* = end-systole, *CMR* = cardiovascular magnetic resonance

Localization of the mitral annular hinge point pairs on both LAx views (referred as mitral landmarks throughout the text) [13] was trained with the same neural network architecture as in module 2. Two comparison metrics were calculated between two independent ratings (specifically, radiologist vs. radiologist or radiologist vs. *Atri-U*): the sum of the Euclidean distances of the mitral landmarks (in mm) and the deviating angle of the mitral annular level (in degrees, for an example see Additional file 1: Figure S3). Upon prediction and in combination with the segmentations from module 2, the longitudinal diameters were automatically placed as the line starting at the mid-point of the mitral annular level (level connecting the two respective mitral landmarks) and passing through the center of mass of the left atrial segmentation [14] (see Fig. 1).

#### Computation of ${LAVI}_{max}$

Each of the above modules was evaluated separately by descriptive statistics, between the two radiologists and between each radiologist and *Atri-U*. Additionally, maximum LA volume index (${LAVI}_{max}$, in mL/m^2^ body surface area) was calculated with the biplane area-length method from the combination of atrial areas and diameters at the detected ES frames (without further modification) and compared between ${LAVI}_{max}^{h}$ and ${LAVI}_{max}^{a}$ and between ${LAVI}_{max}^{h}$ and ${LAVI}_{max}^{Atri-U}$ with Bland–Altman analyses in R (v 3.4.1, blandr package, v 0.5.1) [15], where:

$${LAVI}_{max}^{h}$$
human segmentations and human ES frame selection.
$${LAVI}_{max}^{a}$$
automated segmentations at predefined (human) ES frame.
$$LAVI_{\max }^{Atri - U}$$
automated segmentations at automatically detected ES frame. Note that the ES frame does not necessarily coincide with the visually selected frame.

We estimated the 95% confidence interval from the inter-rater comparisons (sample B) and assessed what percentage of differences in volumes estimated by *Atri-U* and radiologists for sample C were outside the established confidence bounds for ${LAVI}_{max}^{a}$ and $LAVI_{\max }^{Atri - U}$.

## Results

### Evaluation of *Atri-U* in clinical setting

**Fig. 2:**
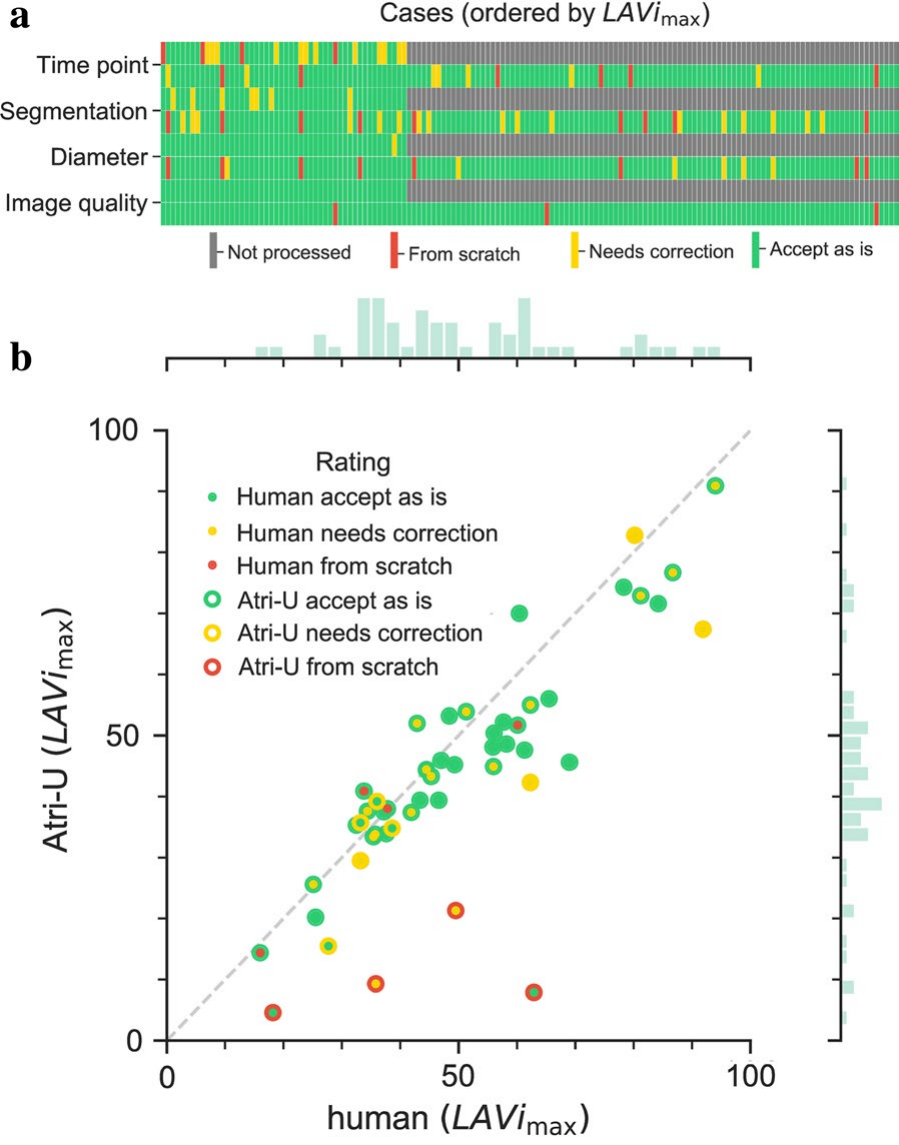
Results from reading of the consecutive clinical sample (samples D_1_ and D_2_). **a** Representation of score received per case, processing step, and processing type (senior radiologist and *Atri-U*, 50 and 150 annotations respectively). The legends are listed at the bottom of the panel. The corresponding proportions are listed in Table 2. **b** Scatter plot of volumes obtained by the senior radiologist (sample D_1_) and *Atri-U* (sample D_2_) for the 50 cases that appeared in both samples, along with their histograms. The color coding corresponds to the highest scores given by the senior cardiologist during the rating of the time-saving potential, where Score 0: 100 percent time saving (no correction needed); Score 1: 50 percent time saving (minor correction needed); and Score 2: no time saving (major correction needed). For example, for the four datapoints with the red rim at the bottom of the graph, he scored the outputs of *Atri-U* as requiring a correction “from scratch”, while for two of them he independently scored the outputs of the senior radiologist as requiring minor correction (yellow center). The majority of cases were scored as requiring no or minor correction and their LAVI_max_ correlate well between human and *Atri-U*. *LAVI*_*max*_ = maximum left atrial volume index

Of the 200 input cines pairs, the cardiologist deemed three as of insufficient image quality for the intended analysis, all belonging to sample D_2_, while in one case the same cine pair was also represented in the D_1_ sample but was there rated as having sufficient quality. According to his scoring, no or minimal correction was required (i) for module 1, in 142 out of 147 cases from *Atri-U* (97%) and 46 out of 50 (92%) from the radiologist, (ii) for module 2 in 138 out of 147 (94%) from *Atri-U* and 50 out of 50 from the radiologist (100%) and (iii) for module 3 in 139 out of 147 (95%, mostly overlapping with interdependent module 2) and 50 out of 50 from the radiologist (100%) (Fig. 2a). While the failure cases for *Atri-U* could be readily identified, the rest of the cases provided a good correlation between manual and automated LA volume estimation (Fig. 2b).

Based on above failure rates and the time to perform the quality assessment and corrections, we estimated the expected average time saving. On average, manual processing of the LA in two views at ES frame would require 5 s for the frame detection, 40 s for segmentation (for 2ch- and 4ch-view, respectively) and 10 s for placement of mitral landmarks/longitudinal diameters (for 2Ch- and 4Ch-view, respectively), in total 105 s. The average time required by the cardiologist for reading the proposed annotations was in average 24.9 and 23.0 s (for samples D_1_ and D_2_, respectively). Thus, the average time required for the complete assessment of the LA using *Atri-U* was 33.9 s (see Table 2). This resulted in a time saving of approximately 71 s per dataset processed by *Atri-U*, which corresponds to a 68 percent reduction compared to the fully manual procedure.

**Table 2:** Estimation of time saving derived from the time-saving proportions, minus the average time required for reading *Atri-U* outputs

	Time required per case (in seconds)	Time saved = manual time $*$ (proportion of data scored as “no need for correction” + 50% $*$ proportion of data scored as “minor corrections needed”)	Proportion of datasets from sample D_2_ for each possible score		
Score			0	1	2^a^
ES frame detection	5	= 5 $*$ 91.3% + 50% $*$ 5 $*$ 4.7% = 4.7 s	91.3%	4.7%	4.0%
Segmentation	80	= 80 $*$ 82.7% + 50% $*$ 80 $*$ 11.3% = 70.7 s	82.7%	11.3%	6.0%
Landmark detection	20	= 20 $*$ 90.7% + 50% $*$ 20 $*$ 5.3% = 18.7 s	90.7%	4.0%	5.3%
Subtotal	105	= 94.1			
Minus reading	23	= 94.1–23.0			
Total time saving		**71.1**			

^a^Score 2 meant “no time saving/correction from scratch”, and therefore is not included in the above calculations; *ES*, End-systolic

### Evaluation of *Atri-U* modules

**Fig. 3:**
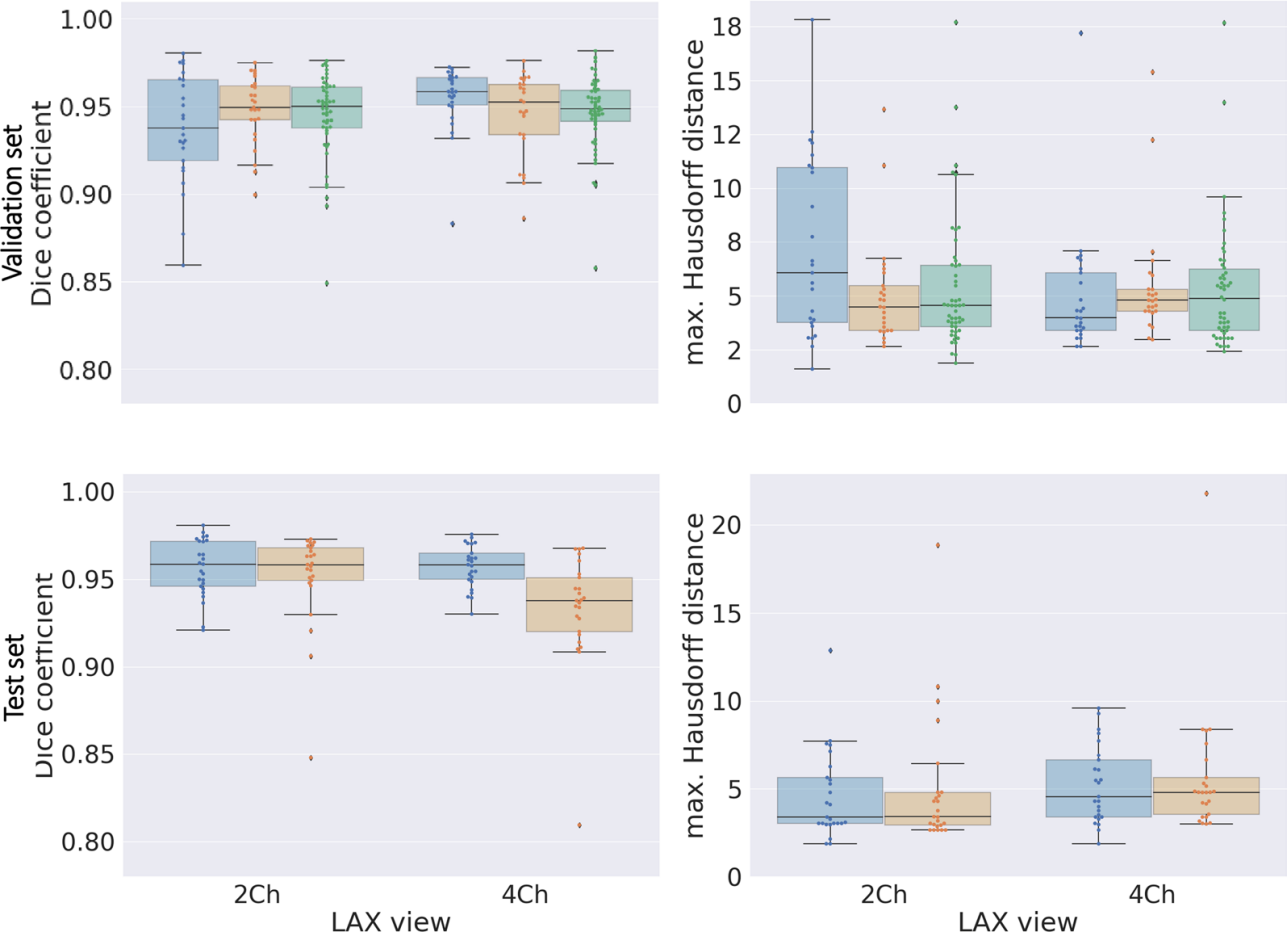
Performance evaluation of segmentation. Boxplots of Dice coefficients and maximum Hausdorff-distance (in mm) values for left atrium area segmentation at end-systole in the validation (sample B) and test (sample C) subsets. In blue radiologist_1_ vs. segmentation algorithm, in orange radiologist_2_ vs. segmentation algorithm and in green radiologist_1_ vs. radiologist_2_. Latter comparison was performed for sample B. The higher variability for sample B compared to C can be explained on the one hand by the difference in sampling type (B mostly included cardiac diseased subjects, while C was random) and on the other hand by the age of the exams: B was selected from older retrospective studies with heterogenous acquisition parameters, while C was selected from a more homogeneous sample acquired within a year. *LAx =* long-axis

In module 1, the median accuracy was 0.96 (Additional file 1: Figure S4 and Table S4). In module 2, the automated LA segmentations had a high overlap of median Dice coefficient of 0.96 and 0.94 with the first radiologist and 0.96 and 0.94 with the second radiologist, for 2Ch- and 4Ch-views respectively (see also Fig. 3 and Additional file 1: Table S5). An overview of the distributions of absolute LA area in the included samples and the inter-rater variability is listed in Additional file 1: Table S6 and S7, respectively. In module 3, median of the sum of distance deviations between radiologist and *Atri-U* for the mitral landmarks was from 4.3 to 6.3 mm, corresponding to a median deviation of 1 to 2.5 pixels per hinge point. *Atri-U* and the radiologists had angle deviations of the mitral annular levels in the same range as inter-rater variability (Additional file 1: Table S8). The resulting lengths of the longitudinal diameters from the combination of LA area segmentations and mitral landmarks are listed in Additional file 1: Table S9. Apart from the variant used by default (i.e. passing through the center of mass of the atrial area), the longitudinal diameters constructed as the longest perpendicular line from the mitral annular level to the posterior wall of the atrium are also listed there.

### ${LAVI}_{max}$ at predefined and at detected ES frame

**Fig. 4:**
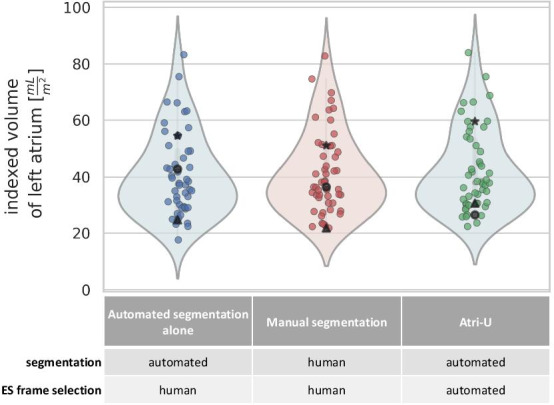
Left atrial volume distributions for combinations of manual and automated steps for segmentation and end-systolic (ES) frame selection. Estimated distribution of maximum LAVI in sample C calculated middle: from human raters, left: by automated segmentation on the same time-frame as for the human rating (LA volumes did not substantially deviate from human) and right: by automated segmentation after additional prediction of the end-systolic (ES) frame (*Atri-U*). With *Atri-U*, 47 cases showed values within the acceptable limits, while 3 cases deviated more (marked as triangles, circles and stars across all three methods). In general, the lower and upper boundaries of the violin plot represent minimum and maximum values, respectively. The distribution of the underlying data (scatter) is represented by the curved sides of the plot

At the visually selected ES frame, the ${LAVI}_{max}^{a}$ values computed from modules 2 and 3 were for all 50 cases of sample C within the previously defined confidence bounds. In the Bland–Altman analyses, there was a slight average underestimation of 0.6 mL/m^2^ (upper and lower limits of agreement of − 4.9 and 6.0, respectively) compared to the calculation based on human segmentations (${LAVI}_{max}^{h}$). In the complete version, where the ES frame was additionally selected automatically $\left( {LAVI_{\max }^{Atri - U} } \right)$, 47 of 50 cases were within the previously defined confidence bounds (94%), with a slight average overestimation of 0.8 mL/m^2^ (upper and lower limits of agreement − 7.5 and 5.8, respectively) (see also Fig. 4 and Additional file 1: Figures S5, S6 and Table S10). The accuracy of volume prediction was independent of LA size. Susceptibility of LA volume to time frame selection is shown in Additional file 1: Figure S7.

### Availability of *Atri-U*

At our institution, *Atri-U* is implemented in the clinical routine, as previously described [16]. For each qualifying CMR study, *Atri-U* is automatically triggered and it takes approximately 120 s until the automatically calculated ${LAV}_{max}$ and ${LAVI}_{max}$ is available.^2^ Since *Atri-U* is not a certified medical product, the segmentations, longitudinal diameters and the predicted ES frame are reviewed for potential errors and, after manual revision by the expert, the volumetric values get updated accordingly (see video in Additional file 2). A stand-alone backend version of *Atri-U* is available on a public repository.

## Discussion

We developed an automated image-analysis pipeline for the computation of ${LAV}_{max}$ analysis from LAx CMR cines and integrated it into an established clinical workflow. The expected average time gain when checking and applying minor corrections instead of annotating from ground-up was substantial. In the exemplary clinical setting, the cardiologist required only one third of the time of the original process for reading automatically generated proposals, while retaining the reliability and accuracy that is required in clinical practice. The cardiologist identified the highest need for manual interaction in the segmentations of atrial areas (six percent of *Atri-U* segmentations), which was the most time-consuming step of the analysis and a strong contributor to the overall time-saving effect of *Atri-U*. The required correction rates for de novo ES frame selection and placement of longitudinal diameters based on mitral landmarks were three and five percent, respectively.

Each step of the biplane area-length method, traditionally being performed by the human expert, was implemented as a fully convolutional neural network module and in general reached human-level performance with sporadic failure cases. *Atri-U* is the first assessment of an automated ${LAV}_{max}$ estimation from CMR cines, meeting the requirements for clinical use as presented in the introduction. *Atri-U* is also filling the gap of fully automated processing of bulk datasets for research, as it can be used, retrained, and even adapted and extended independently of the user interface, unlike commercially available tools.

Fully convolutional neural network segmentation was recently introduced for LAx views of the LA in patients with hypertrophic cardiomyopathy [17]. In contrast to this rather homogenous population, the presented algorithms were trained on and evaluated with heterogenous samples consisting of routine CMR cines originating from patients with a variety of heart diseases, including atrial fibrillation. Irrespective of the size of the LA, Dice coefficients of around 0.95 were obtained, similar to a study with almost 4000 training cases [12]. Narrower limits of agreement have been previously published for LA segmentation [18], yet on healthy subjects, while in our samples—extracted from the hospital database—all grades of atrial dilatation were represented [19]. Unlike segmentation, the detection of the relevant ES frame and the mitral landmarks are two mostly unexplored [13] but essential steps in order to further automate LA volume estimation with the biplane area-length method. We measured the LA longitudinal diameter and by extension the estimated LA volume in two ways, as either the line passing through the center of mass or as the longest perpendicular line passing through the mitral annulus [14]. This option for comparison of the two approaches shows the flexibility of our clinically embedded software development platform, where different calculation methods or even a manual editing of the diameter can be readily implemented on top of *Atri-U*.

We did not only evaluate the performance of single steps of the pipeline (ES frame detection, segmentation of the LA in each cine view, localization of the mitral landmarks), but also the combination thereof. Due to the propagation of variance from one step to the other, we observed larger deviations from human ground-truth when the automated detection of the ES frame was added to the process. Although in three out of 50 cases this resulted in a predicted ${LAV}_{max}$ outside the previously defined confidence bounds (sample C), the variability in ES frame selection was also observed between the two experts (sample D_1_).

While the focus of our study was on 2D LAx CMR, obtaining the volume directly from the segmentation of the LA in multislice stacks is of potential clinical interest [20]. With the same fully convolutional neural networks model as for LAx cines, but adapted to incorporate context along the third dimension, we could show human-level performance in the segmentation of the LA in 3D cines for all phases of the cardiac cycle, in cases with atrial fibrillation and/or LA enlargement, showing a strong correlation with 2D volumetry (see Additional file 1: Sections E3, E4 and video in Additional file 3). In such an application, the time gain may be even higher than for 2D image analysis. Both the 3D and the 2D quantifications could also be useful to assess longitudinal changes in LA volumetry.

Out of the recently suggested nine key considerations for study design in artificial intelligence image analysis [21], six items were fulfilled and three were not: as a limitation, we did not include a fully independent external test subset (consideration 2) and did not include multi-vendor image acquisitions (consideration 3). Multiple devices, sequences and magnetic field strengths were used, despite acquisition with equipment of the same manufacturer. The third consideration that was not fulfilled is to demonstrate the way the algorithm makes the decision (consideration 8). Continuous application of *Atri-U* will inform about the potential of the method and possibly reveal failure cases that can be addressed by relatively trivial re-training of the model. The six key considerations that our study fulfilled include: the split of the sample into training, validation, and test subsamples (consideration 1) of appropriate size (consideration 4). *Atri-U* was trained using manual annotation (consideration 5) and the performance of *Atri-U* was compared to that of radiology experts (consideration 7), albeit not with a statistical test. By publishing code and models (consideration 9) that operate on the raw images (consideration 6), we facilitate objective comparison with alternative methods and evaluation on other (external) datasets. Because of the considerations that were not met, we cannot conclude about the generalizability of the method with the pretrained models in any other case than our local. Additionally, it assumes that placement of the LAx cines is in conformity with the current guidelines [4], since slice positioning might impact the accuracy of LA volume quantification and lead to differences in volume estimation from exam to exam. Finally, the design of the study did not allow testing for non-inferiority of the proposed method. However, the intended application is integrated in a pipeline in which experts validate every single processing step and systematic failure in a new setting would be readily recognizable.

## Conclusions

We have developed, evaluated, and deployed a process for automated calculation of ${LAV}_{max}$ from LAx CMR cines that offers an average time saving for evaluation of clinical CMR exams of more than a minute per case (66% reduction). The integration into an existing clinical workflow provides options for review and revision of individual steps, ensuring that the expert retains control over the process and the clinical end-point. As the LAx cines are part of a whole-heart CMR protocol, their automated segmentation, including the calculation of ${LAV}_{max}$, further contributes to the comprehensive analysis of all four heart chambers. The broad availability of automated segmentation and volumetry will greatly facilitate the analysis of quantitative parameters of the LA in clinical settings as well as in research studies of cardiac diseases.

## Supplementary Information

**Additional file 1.** Contains descriptions of the studied populations, complementary results on segmentation and mitral valve classification, as well as detailed description of methods used for training and evaluation of the algorithms. Finally, a 3D cine segmentation algorithm is presented in detail.

**Additional file 2.** A video recording of the implementation of *Atri-U* in the clinical workflow established at our institution. Please note that *Atri-U* is not a certified medical product.

**Additional file 3.** An exemplary result is depicted on a 3D stack video recording. 3D cine segmentation of the left atrium (LA) at three distinct phases of the cardiac cycle: end-systole (left), end-diastole (middle) and directly preceding atrial contraction (right). The manual segmentation is shown in red and the automated segmentation in blue.

## Data Availability

The imaging datasets underlying this article are not publicly available due to their containing information that could compromise the privacy of participants. However, software code generated during this study, a stand-alone backend version of *Atri-U* with trained models, and a sample dataset will be made available on a public repository: https://git.upd.unibe.ch/openscience/atri-u; Operating system: Platform independent; Programming languages: Python, Matlab; Required packages: NiftyNet; License: GNU General Public License v3.0.

## Abbreviations

CMR: Cardiovascular magnetic resonance
ES: End-systolic
LA: Left atrium/left atrial
*LAV*_*max*_: Maximum left atrial volume
*LAVI*_*max*_: Maximum left atrial volume index
LAx: Long axis
2Ch: Two-chamber view
4Ch: Four-chamber view

## Footnotes

1 All references to the time point of the cardiac cycle are made with respect to the ventricular cardiac phase.

2 This is the time that passes from the acquisition until the results are available to the radiologist. The time needed by the fully convolutional neural networks for the image analysis is less than five seconds. The rest of the time is consumed by data transfer over the network and format conversions. The body surface area is calculated from the image file-header fields for subject’s weight and height.
