# Supplementary material for: Atri-U: assisted image analysis in routine cardiovascular magnetic resonance volumetry of the left atrium
Source: J Cardiovasc Magn Reson. 2021 Nov 11;23:133. doi: 10.1186/s12968-021-00791-8 (PMC8582149; doi:10.1186/s12968-021-00791-8)

Additional file 1

Index

[Section E1](#_Toc71659804)

[Dataset 2](#_Toc71659805)

[Exclusion/selection criteria and sampling process 2](#_Toc71659806)

[Acquisition parameters of long-axis (LAx) cines 3](#_Toc71659807)

[Section E2](#_Toc71659808)

[Manual annotation 19](#_Toc71659809)

[Deep learning algorithms and post-processing methods 19](#_Toc71659810)

[Computation of left atrial volume from the 2D cines 22](#_Toc71659811)

[Section E3 - 3D cines](#_Toc71659812)

[Dataset 23](#_Toc71659813)

[Manual contouring 24](#_Toc71659814)

[Automated segmentation 24](#_Toc71659815)

[Computed left atrial volume from the 3D cines 26](#_Toc71659816)

[Section E4](#_Toc71659817)

[Dataset 29](#_Toc71659818)

[2D/3D correlation 29](#_Toc71659819)

# Section E1

## Dataset

## Exclusion/selection criteria and sampling process

Supplementary Table S1 and Supplementary Figure S1 show the step-wise selection, including the following:

- initial exclusion of 215 datasets from subjects who did not consent to the study, subjects younger than 18 years and subjects with missing cine data
- a further exclusion of 103 subjects based on clinical and image quality parameters
- The then remaining datasets of 1379 (N=81.3%) subjects were divided in two periods of image acquisitions (01/2014 until 06/2018 and 07/2018 until 06/2019, respectively).

| **Supplementary Figure S1. Criteria for data selection and composition of subset samples.** Flowchart of the selection and sampling process (values represent counts of subjects). ^1^ low image quality. ^2^ for details see Supplementary Table S1. ^3^ ratio of magnetic field strengths (1.5:3.0 Tesla). For 3D cines see in the last section of supplementary materials |
| --- |


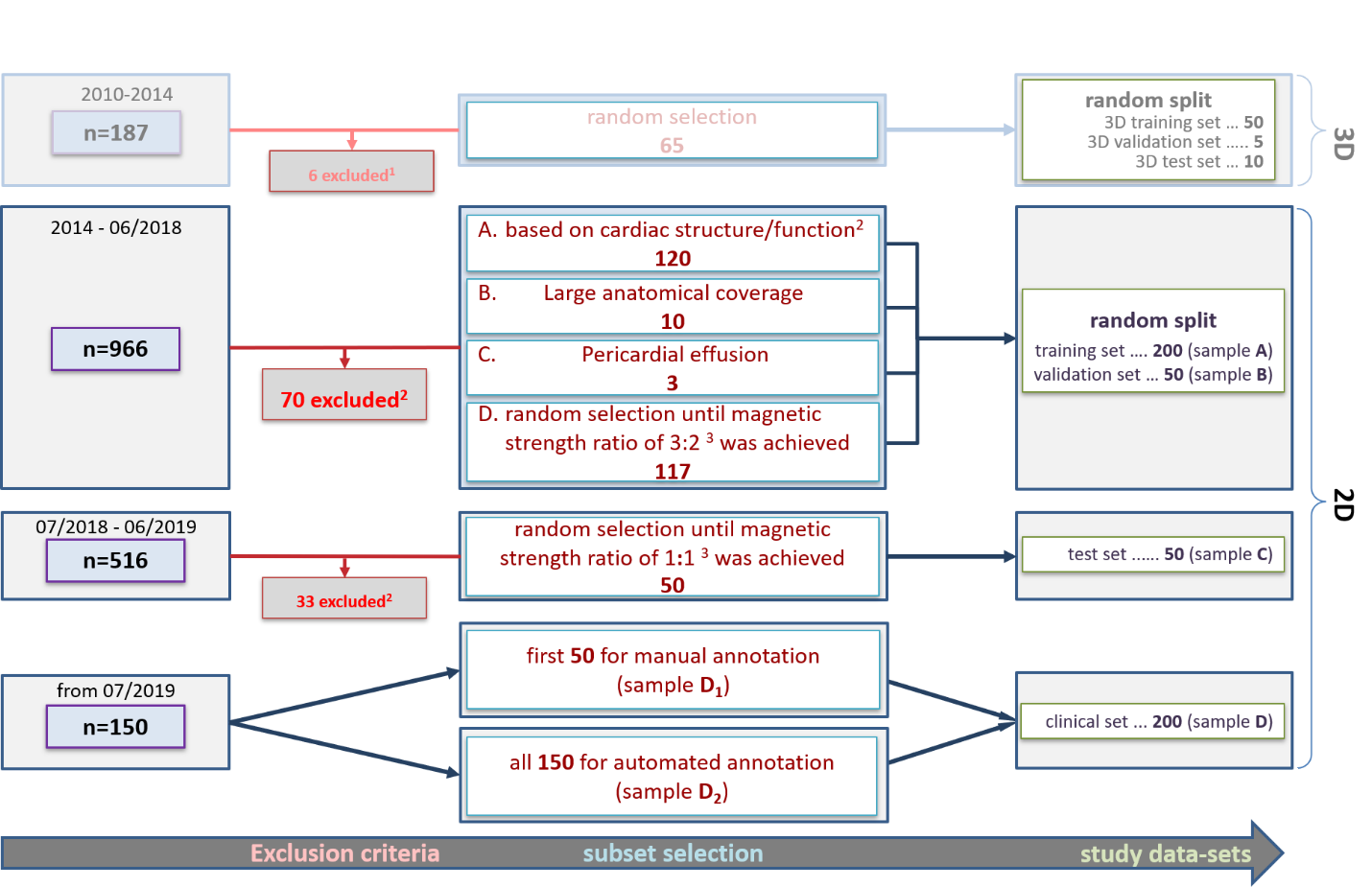


- Subsets of 2ch- and 4ch-view pairs from the acquisitions recorded from 2014 until the first half of 2018 were selected for training (sample A, $N=200$) and validation (sample B, $N=50$) of the neural networks, in part based on established volumetric thresholds for the cardiac chambers set by a recent cardiac segmentation benchmark study (N=120, see Supplementary Table S1), presence of pericardial effusion (N=3) and large field of view (N=10) and in part randomly (N=117). An independent sub-sample from the last year of recordings provided the source for the test-set (sample C, $N=50$ randomly selected cine pairs).

## Acquisition parameters of long-axis (LAx) cines

CMR examinations had been performed between 2014 and June 2019 in 1697 patients (100%) on different magnetic field strengths (1.5 Tesla: Magnetom Espree or Magnetom Avanto, 3.0 Tesla: Verio or Magnetom Skyra, Siemens, Erlangen, Germany) with balanced steady-state-free precession (b-SSFP) sequences and retrospective ECG gating. The cardiac cycle of all participants was sampled with 25 time-frames per cycle in both views. As the single-slice two- and four-chamber long-axis cines were part of the clinical routine CMR protocol, the acquisition parameters acquired over a period of 5 years had been determined by the radiological staff and were not harmonized over the cases. Placement of cardiac views was proposed by a dedicated vendor-specific planning software (*Cardiac Dot Engine*, Siemens Healthineers) installed on the scanner and confirmed by the radiological staff. For the two-chamber view, acquisition matrix ranged between 138x192 and 256x256 and pixel size between 1.25x1.25 and 2.2x2.2 mm^2^. For the four-chamber view, acquisition matrix was 150x192 to 256x256 and pixel size 1.25 x 1.25 to 2.3x2.3 mm^2^. The slice thickness was 6 mm for both views. Additionally to the N=1697, consecutively acquired studies were collected for the final step of validation on a clinical sample, starting July 2019 and until N=150 were completed (forming samples D_1_/D_2_).

**Supplementary Table S1. Exclusion and selection criteria for the long-axis cine datasets from a total of** $\boldsymbol{N=1697}$ **(100%)**

|  |  | **Training and validation** | |  |  | **Test** |  | | **Total (%)** | | | |  |
| --- | --- | --- | --- | --- | --- | --- | --- | --- | --- | --- | --- | --- | --- |
| **EXCLUSION CRITERIA** | **1. Formal exclusion criteria** | | | | | | | | | | |  |  |
|  | No consent | | 58 |  |  | 33 |  |  | | 91 (5.4%) | | | |
|  | Underage (<18 years) | | 13 |  |  | 4 |  |  | | 17 (1.0%) | | | |
|  | Missing data | | 90 |  |  | 17 |  |  | | 107 (6.3%) | | | |
|  | **Subtotal** | | **161** |  |  | **54** |  |  | | **215** | | | |
|  | **2. Second exclusion step, based on image-based and clinical reports** | | | | | | | | | |  |  |  |
|  | Low image quality | | 22 |  |  | 4 |  |  | | 26 (1.5%) | | | |
|  | Congenital disease | | 31 |  |  | 16 |  |  | | 47 (2.8%) | | | |
|  | Valve repair or calcification | | 9 |  |  | 7 |  |  | | 16 (0.9%) | | | |
|  | Cardiac tumor | | 8 |  |  | 6 |  |  | | 14 (0.8%) | | | |
|  | **Subtotal** | | **70** |  |  | **33** |  |  | | **103** | | | |
| **SUBSET SELECTION** | **3. Clinical selection criteria for the training and validation subsets**, adapted from a recent benchmark study* | | | | | | | | | | |  |  |
|  | **“Normal”**  LV-EF ≥ 55% AND RV-EF > 40% AND RV-EDVi < 100 ml/m^2^ AND myocardial wall-thickness < 15 mm AND (for males LV-EDVi < 90 ml/m^2^ OR for females LV-EDVi < 80 ml/m^2^) | | 20 |  |  | - |  |  | |  | | | |
|  | **“Myocardial infarction”**  LV-EF ≤ 40% AND reported wall motion abnormalities | | 20 |  |  | - |  |  | |  | | | |
|  | **“Dilative cardiomyopathy”**  LV-EF ≤ 40% AND LV-EDVi ≥ 100 mL/m2 AND no reported wall motion abnormalities | | 20 |  |  | - |  |  | |  | | | |
|  | **“Hypertrophic cardiomyopathy”**  LV-EF ≥ 55% AND myocardial wall thickness ≥ 15 mm | | 20 |  |  | - |  |  | |  | | | |
|  | **“Abnormal right ventricle”**  RV-EF ≤ 40 % AND (for males RV-EDVi > 110 mL/m^2^ OR for females RV-EDVi > 100 mL/m^2^) | | 20 |  |  | - |  |  | |  | | | |
|  | **not fulfilling any of the published criteria**  Neither belonging to the normal, nor to a pathological group | | 20 |  |  | - |  |  | |  | | | |
|  | *** Bernard O, Lalande A, Zotti C, Cervenansky F, Yang X, Heng PA, et al. Deep learning techniques for automatic MRI cardiac multi-structures segmentation and diagnosis: is the problem solved? IEEE Trans Med Imaging. 2018;37:2514–25. Abbreviations*: LV: Left ventricle, RV: right ventricle, EF: ejection fraction, EDVi: indexed end-diastolic volume. Capital AND/OR represent logical conjunctions.* | | | | | | | | | | |  |  |

**Supplementary Table S2. Basic demographic and clinical characteristics of the population at baseline by** **sample**

|  | **sample A** |  | **sample B** |  | **sample C** |  | **sample D_1_/D_2_** |
| --- | --- | --- | --- | --- | --- | --- | --- |
| **Sample size** | **200** |  | **50** |  | **50** |  | **150** |
| **Age [years]**; median (range) [q1, q3] | 57 (18–90)  [46, 67] |  | 53 (19–78)  [42, 62] |  | 58 (19–82)  [46, 67] |  | 60 (19–82)  [48, 70] |
| **Sex**; male/female | 164 / 86 |  | 30 / 20 |  | 35 / 15 |  | 99 / 51 |
| **Body surface area [m^2^]**; median (range) [q1, q3] | 1.93 (1.38–2.68)  [1.77, 2.06] |  | 1.97 (1.57–2.43)  [1.81, 2.12] |  | 1.95 (1.44–2.35)  [1.78, 2.09] |  | 1.88 (1.35–2.38)  [1.71, 2.06] |
| **Reported findings*** |  |  |  |  |  |  |  |
| **Atrial dilatation** | 52 (26%) |  | 12 (24%) |  | 16 (32%) |  | 30 (20%) |
| - left | 15 |  | 6 |  | 7 |  | 17 |
| - right | 4 |  | 3 |  | 3 |  | 1 |
| - both | 33 |  | 3 |  | 6 |  | 13 |
| **Mitral valve insufficiency** | 58 (29%) |  | 22 (44%) |  | 13 (26%) |  | 39 (26%) |
| **Structural heart disease** | 151 (76%) |  | 38 (76%) |  | 35 (70%) |  | 103 (69%) |
| - ischemic | 44 |  | 8 |  | 10 |  | 32 |
| - Non-ischemic cardiomyopathies | 77 |  | 20 |  | 13 |  | 55 |
| - Valvular | 4 |  | 2 |  | 1 |  | 6 |
| - structural findings, no conclusive disease | 26 |  | 8 |  | 11 |  | 10 |
| **No structural findings** | 49 (25%) |  | 12 (24%) |  | 15 (30%) |  | 47 (31%) |
| **Magnetic field strength** (1.5/3.0 Tesla) | 117/83 |  | 33/17 |  | 25/25 |  | ­­­112/38 |
| *as extracted from written cardiac magnetic resonance reports. Findings listed are not mutually exclusive. Abbreviations: q1/q3: 1^st^/ 3^rd^ quartile | | | | | | | |

***Supplementary Figure S2. Detailed listing of the cardiac magnetic resonance diagnosis as extracted from the radiological report accompanying the LAx cines, separately for all four subsamples*** *(A - D) (see also Supplementary Table S2). Findings are not mutually exclusive.*

where AF: atrial fibrillation, AIC: arrhythmia-induced cardiomyopathy, Am: amyloidosis, ARVC: arrhythmogenic right ventricular cardiomyopathy, AC: autoimmune cardiopathy, CS: cardiac sarcoidosis, DCM: dilative cardiomyopathy, HCM: hypertrophic cardiomyopathy, HTCM: hypertensive cardiomyopathy, isc: ischemia without infarction, MI: myocardial infarction, MC: myocarditis/perimyocarditis, MDC: muscular dystrophy-related cardiopathy, NCC: Non-compaction cardiomyopathy, TS: Tako-Tsubo and VC: valvular cardiopathy, nc: structural findings but no conclusive disease, nsf: no structural findings. In sample D “other” (n=6) include iron-overload (3), alcohol-induced cardiopathy (1), drug-induced cardiopathy (1), congenital cardiac anomaly (1).

**Supplementary Table S3. Established evaluation metrics for comparison between the segmentations**

| **Metric** | **Formula** | **Definition** |
| --- | --- | --- |
| **Dice similarity coefficient** | $\frac{2\vert A\cap M\vert}{\vert A\vert\cup\vert M\vert}$ | twice the number of pixels/voxels common to both the A* and M† segmentations divided by the sum of number of pixels/voxels in each segmentation. Ranges from 0 to 1. |
| **Jaccard index** | $\frac{\vert A\cap M\vert}{\vert A\cup M\vert}$ | intersection divided by the size of the union. Ranges from 0 to 1. |
| **Pixelwise recall** | $\frac{\vert A\cap M\vert}{\vert M\vert}$ | number of pixels/voxels common to both segmentations divided by number of pixels/voxels in the manual segmentation. Ranges from 0 to 1. |
| **Pixelwise precision** | $\frac{\vert A\cap M\vert}{\vert A\vert}$ | number of pixels/voxels common to both segmentations divided by number of pixels/voxels in the automated segmentation. Ranges from 0 to 1. |
| **Absolute relative area difference** | $\frac{\vert A-M\vert}{\vert M\vert}$ | number of pixels/voxels of manual minus number of pixels/voxels in the automated segmentation, divided by number of pixels/voxels of manual segmentation. Ranges from 0 to 1. |
| **Maximal Hausdorff distance** | $max(\underset{p\in\partial A}{max d} (p,\partial M),\underset{p\in\partial M}{max d} \left( p,\partial A \right))$  where d(p,∂) denotes the minimal distance from point $p$ to contour $\partial$ | The maximum distance  between the segmentation contours $\partial A$ and $\partial M$ in mm. |
| ** A is the automated segmentation, † M is the manual segmentation* | | |

***Supplementary Figure S3. Comparison of mitral landmarks localization.*** *Performance evaluation of the mitral landmarks (anterior and inferior in the case of a 2ch-view) with definition of two metrics: sum of distance deviation (d_a_+d_i_, in mm) and angle deviation (φ) between two segmentation pairs (orange and yellow, respectively)*


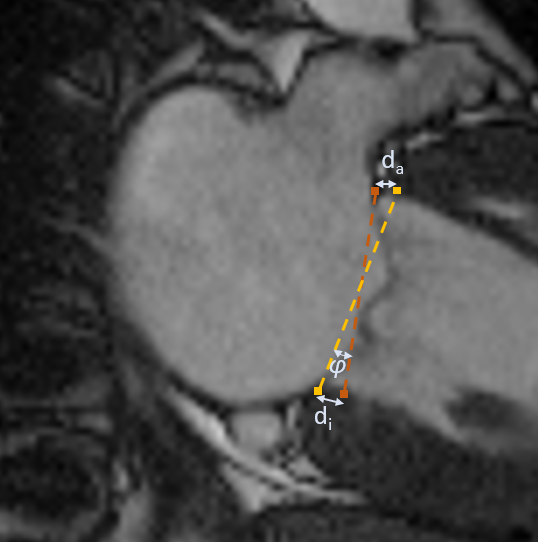


**Supplementary Table S4. Comparison metrics for the mitral state predictions** **in the 4ch view.** A convolutional neural network was trained for the prediction of mitral valve states, based on reference classifications for each four-chamber frame. Comparison metrics for mitral valve states in the 4ch-view for samples B (N=50) and C (N=50). Accuracy is per case, while precision and recall refer to the closed state. For both samples the end-systolic time frame was detected with a high accuracy (median 0.96), with deviations from the reference annotation of maximum 2 frames and 1 frame, respectively (see also Figure S4)

|  |  | **sample B (validation subset)** | | |  | **sample C (test subset)** | | |
| --- | --- | --- | --- | --- | --- | --- | --- | --- |
|  |  | **accuracy** | **Precision (closed)** | **Recall (closed)** |  | **accuracy** | **precision (closed)** | **recall (closed)** |
| **median** |  | 0.96 | 0.96 | 0.93 |  | 0.96 | 1.00 | 0.92 |
| **worst** |  | 0.76 | 0.76 | 0.70 |  | 0.80 | 0.77 | 0.50 |
| **worse quartile** |  | 0.88 | 0.88 | 0.88 |  | 0.92 | 0.94 | 0.90 |
| **better quartile** |  | 1.00 | 1.00 | 1.00 |  | 0.97 | 1.00 | 1.00 |
| **best** |  | 1.00 | 1.00 | 1.00 |  | 1.00 | 1.00 | 1.00 |
| **average** |  | 0.94 | 0.94 | 0.93 |  | 0.94 | 0.97 | 0.91 |
| **SD** |  | 0.06 | 0.06 | 0.08 |  | 0.05 | 0.06 | 0.11 |
| *The notations “worst” to “best” refer to the performance of the comparisons and correspond to minimum, quartiles and maximum for all columns, except for columns “absolute relative area difference” and “max. Hausdorff distance”, where the order from min to max is descending. SD=one standard deviation.* | | | | | | | | |

**Supplementary Figure S4. Detection of frame at ventricular end-systole from the classification of mitral valve state.** Prediction of mitral valve state in sample B and C (the validation and test set, respectively) on each frame of the four-chamber cines (50 cases each). Each row represents one case with 25 columns corresponding to the individual frames. Typically, the cines start at end-diastole and the first frames show a closed valve state (orange colors), followed by frame blocks of open valve states (blue colors). The light colors show false positive and negative predictions, respectively. Results show excellent prediction of mitral valve state. After prediction the end-systolic time-frame is being selected as the last frame of the largest block of frames with closed mitral valve. For sample B 32 cases showed an overlap, 16 cases a deviation of ±1 and 2 cases of ±2, while for sample C 33 cases showed no deviation and 17 a deviation of ±1 frame.

| 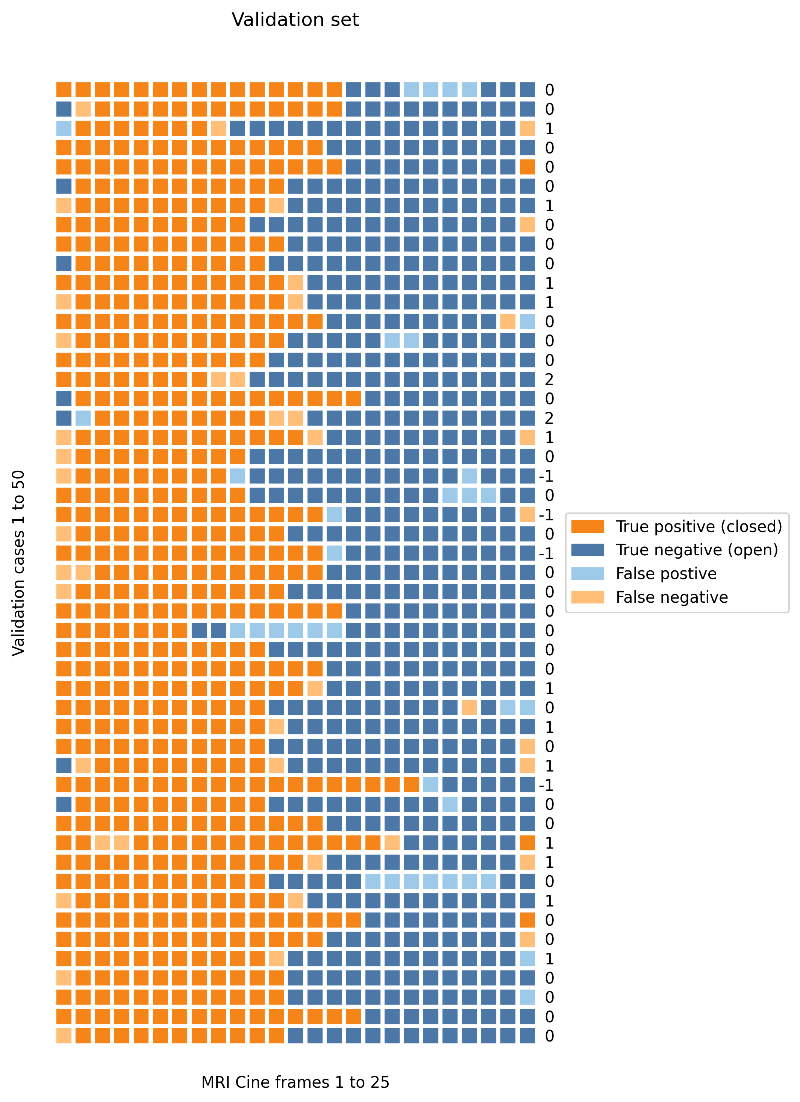 | 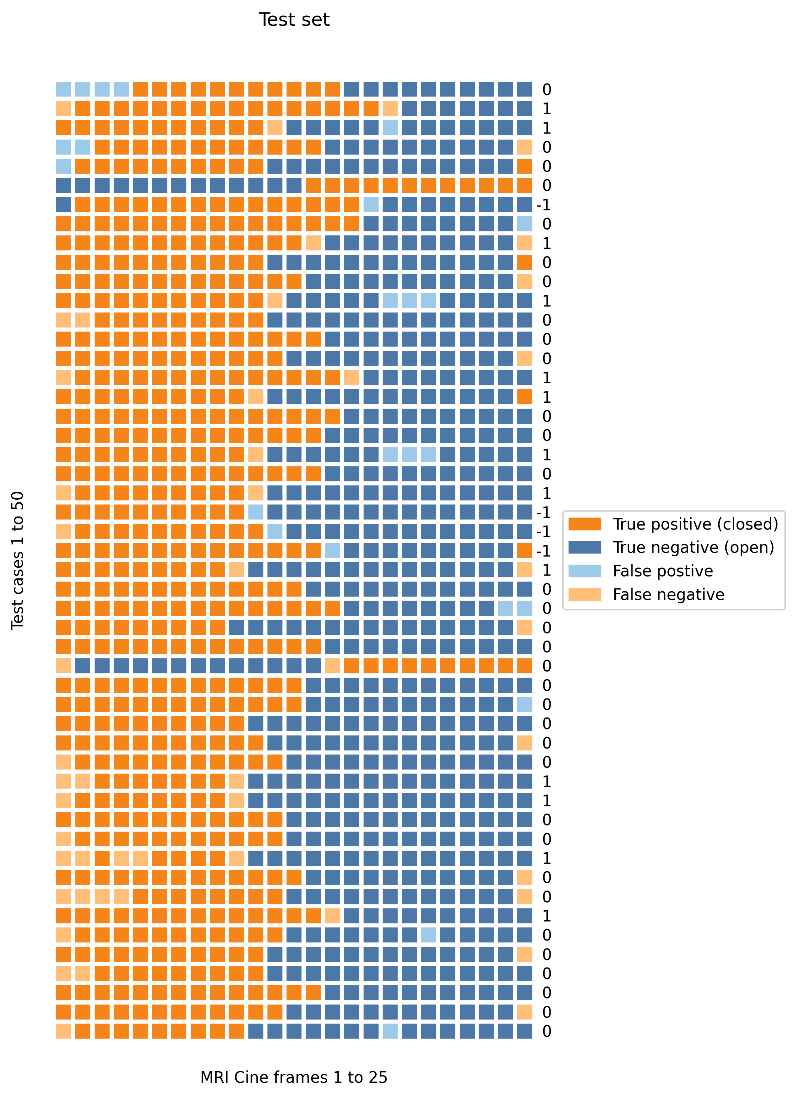 |
| --- | --- |

***Supplementary Table S5. Performance evaluation for the left atrial area segmentation in sample C***

|  | **Dice coefficient** | | **Jaccard index** | | **Pixelwise precision** | | **Pixelwise recall** | | **Absolute relative area difference** | | **Max. Hausdorff distance (mm)** | |
| --- | --- | --- | --- | --- | --- | --- | --- | --- | --- | --- | --- | --- |
|  | **2ch** | **4ch** | **2ch** | **4ch** | **2ch** | **4ch** | **2ch** | **4ch** | **2ch** | **4ch** | **2ch** | **4ch** |
| **median rater_1_ vs. algorithm** | 0.96 | 0.96 | 0.92 | 0.92 | 0.97 | 0.96 | 0.95 | 0.96 | 0.02 | 0.03 | 3.4 | 4.6 |
| **median rater_2_ vs. algorithm** | 0.96 | 0.94 | 0.92 | 0.88 | 0.97 | 0.91 | 0.95 | 0.97 | 0.03 | 0.06 | 3.4 | 4.8 |
| **worst rater_1_ vs. algorithm** | 0.92 | 0.93 | 0.85 | 0.87 | 0.91 | 0.89 | 0.88 | 0.90 | 0.09 | 0.09 | 12.9 | 9.6 |
| **worst rater_2_ vs. algorithm** | 0.85 | 0.81 | 0.74 | 0.68 | 0.85 | 0.69 | 0.76 | 0.93 | 0.22 | 0.43 | 18.9 | 21.8 |
| **worse quartile rater_1_ vs. algorithm** | 0.95 | 0.95 | 0.90 | 0.90 | 0.95 | 0.93 | 0.94 | 0.95 | 0.04 | 0.05 | 5.6 | 6.6 |
| **worse quartile rater_2_ vs. algorithm** | 0.95 | 0.92 | 0.90 | 0.85 | 0.95 | 0.89 | 0.94 | 0.96 | 0.04 | 0.11 | 4.8 | 5.6 |
| **better quartile rater_1_ vs. algorithm** | 0.97 | 0.96 | 0.95 | 0.93 | 0.97 | 0.98 | 0.97 | 0.97 | 0.01 | 0.02 | 3.0 | 3.4 |
| **better quartile rater_2_ vs. algorithm** | 0.97 | 0.95 | 0.94 | 0.91 | 0.97 | 0.94 | 0.96 | 0.98 | 0.02 | 0.03 | 2.9 | 3.6 |
| **best rater_1_ vs. algorithm** | 0.98 | 0.98 | 0.96 | 0.95 | 0.99 | 0.98 | 0.99 | 1.00 | 0 | 0 | 1.9 | 1.9 |
| **best rater_2_ vs. algorithm** | 0.97 | 0.97 | 0.95 | 0.94 | 0.99 | 0.97 | 0.98 | 0.99 | 0.01 | 0 | 2.7 | 3.0 |
| **average rater_1_ vs. algorithm** | 0.96 | 0.96 | 0.92 | 0.92 | 0.96 | 0.95 | 0.95 | 0.96 | 0.03 | 0.04 | 4.6 | 5.2 |
| **average rater_2_ vs. algorithm** | 0.95 | 0.93 | 0.91 | 0.88 | 0.96 | 0.90 | 0.95 | 0.97 | 0.04 | 0.08 | 5.0 | 5.7 |
| **SD rater_1_ vs. algorithm** | 0.02 | 0.01 | 0.03 | 0.02 | 0.02 | 0.03 | 0.03 | 0.02 | 0.03 | 0.03 | 2.5 | 2.2 |
| **SD rater_2_ vs. algorithm** | 0.03 | 0.03 | 0.05 | 0.05 | 0.03 | 0.06 | 0.04 | 0.02 | 0.05 | 0.08 | 3.7 | 3.8 |
| *Note that sample C (N=50) was evenly split for segmentation by the two raters on the end-systolic frame. The notations “worst” to “best” refer to the performance of the comparisons and correspond to minimum, quartiles and maximum for all columns, except for columns “absolute relative area difference” and “max. Hausdorff distance”, where the order from minimum to maximum is descending. The color coding of comparisons corresponds to that of Figure 3 in the main manuscript. Abbreviations = SD: one standard deviation, 2ch/4ch: two-/four-chamber view* | | | | | | | | | | | | |

**Supplementary Table S6. Absolute left atrium area in cm^2^ at (ventricular) end-systolic frame for 2ch- and 4ch-views.** Descriptive statistics of absolute values of left atrial size from manual segmentations for each view by sample, giving an overview of the absolute area distributions. The included datasets show a wide distribution of left atrial size and various grades of left atrial enlargement are represented in all three respective samples.

|  |  | **Training (sample A)** | |  | **Validation (sample B)** | |  | **Test (sample C)** | |
| --- | --- | --- | --- | --- | --- | --- | --- | --- | --- |
| **view** |  | **2ch** | **4ch** |  | **2ch** | **4ch** |  | **2ch** | **4ch** |
| **Subjects (N)** |  | 200 | 200 |  | 50 | 50 |  | 50 | 50 |
| **median** |  | 20.9 | 25.1 |  | 23.5 | 25.6 |  | 19.2 | 22.6 |
| **minimum** |  | 8.4 | 11.6 |  | 12.0 | 12.6 |  | 10.1 | 13.4 |
| **1^st^ quartile** |  | 17.5 | 21.7 |  | 21.6 | 20.2 |  | 16.1 | 20.0 |
| **median** |  | 20.9 | 25.1 |  | 23.5 | 25.6 |  | 19.2 | 22.6 |
| **3^rd^ quartile** |  | 25.4 | 30.5 |  | 25.3 | 28.2 |  | 22.3 | 27.3 |
| **maximum** |  | 66.1 | 67.5 |  | 32.1 | 38.6 |  | 34.5 | 38.4 |
| **average** |  | 22.2 | 26.5 |  | 23.2 | 24.9 |  | 20.0 | 23.9 |
| **SD** |  | 7.3 | 7.7 |  | 4.4 | 5.9 |  | 5.5 | 6.1 |
| *For* *both long-axis views of sample A, the first rater respectively was taken into account. SD=one standard deviation.* | | | | | | | | | |

**Supplementary Table S7. Performance evaluation of left atrial area segmentation on sample B (N=50).** The two human raters showed a high overlap of left atrial segmentations in end-systole with a median Dice coefficient of 0.95 for both the 2ch- and 4ch-view.

|  | **Dice coefficient** | | **Jaccard index** | | **Pixelwise precision** | | **Pixelwise recall** | | **Abs. relative area difference** | | **Max. Hausdorff distance (mm)** | |
| --- | --- | --- | --- | --- | --- | --- | --- | --- | --- | --- | --- | --- |
|  | **2ch** | **4ch** | **2ch** | **4ch** | **2ch** | **4ch** | **2ch** | **4ch** | **2ch** | **4ch** | **2ch** | **4ch** |
| **median rater_1_ vs. rater_2_** | 0.95 | 0.95 | 0.90 | 0.90 | 0.96 | 0.94 | 0.95 | 0.97 | 0.04 | 0.05 | 4.5 | 4.9 |
| **median rater_1_ vs. algorithm** | 0.94 | 0.96 | 0.88 | 0.92 | 0.93 | 0.96 | 0.97 | 0.96 | 0.07 | 0.02 | 6.1 | 4.0 |
| **median rater_2_ vs. algorithm** | 0.95 | 0.95 | 0.90 | 0.91 | 0.95 | 0.94 | 0.96 | 0.98 | 0.04 | 0.06 | 4.5 | 4.8 |
| **worst rater_1_ vs. rater_2_** | 0.85 | 0.86 | 0.74 | 0.75 | 0.82 | 0.84 | 0.74 | 0.87 | 0.33 | 0.18 | 17.7 | 17.7 |
| **worst rater_1_ vs. algorithm** | 0.86 | 0.88 | 0.75 | 0.79 | 0.76 | 0.89 | 0.88 | 0.87 | 0.31 | 0.07 | 17.8 | 17.2 |
| **worst rater_2_ vs. algorithm** | 0.90 | 0.89 | 0.82 | 0.80 | 0.87 | 0.80 | 0.88 | 0.88 | 0.12 | 0.25 | 13.7 | 15.4 |
| **worse quartile rater_1_ vs. rater_2_** | 0.94 | 0.94 | 0.88 | 0.89 | 0.93 | 0.91 | 0.94 | 0.95 | 0.07 | 0.07 | 6.4 | 6.2 |
| **worse quartile rater_1_ vs. algorithm** | 0.92 | 0.95 | 0.85 | 0.91 | 0.88 | 0.95 | 0.96 | 0.95 | 0.11 | 0.03 | 10.9 | 6.1 |
| **worse quartile rater_2_ vs. algorithm** | 0.94 | 0.93 | 0.89 | 0.88 | 0.93 | 0.91 | 0.94 | 0.95 | 0.07 | 0.08 | 5.5 | 5.3 |
| **median rater_1_ vs. rater_2_** | 0.95 | 0.95 | 0.90 | 0.90 | 0.96 | 0.94 | 0.95 | 0.97 | 0.04 | 0.05 | 4.5 | 4.9 |
| **median rater_1_ vs. algorithm** | 0.94 | 0.96 | 0.88 | 0.92 | 0.93 | 0.96 | 0.97 | 0.96 | 0.07 | 0.02 | 6.1 | 4.0 |
| **median rater_2_ vs. algorithm** | 0.95 | 0.95 | 0.90 | 0.91 | 0.95 | 0.94 | 0.96 | 0.98 | 0.04 | 0.06 | 4.5 | 4.8 |
| **better quartile rater_1_ vs. rater_2_** | 0.96 | 0.96 | 0.92 | 0.92 | 0.97 | 0.97 | 0.97 | 0.98 | 0.01 | 0.02 | 3.6 | 3.4 |
| **better quartile rater_1_ vs. algorithm** | 0.97 | 0.97 | 0.93 | 0.93 | 0.97 | 0.97 | 0.98 | 0.97 | 0.02 | 0.01 | 3.8 | 3.4 |
| **better quartile rater_2_ vs. algorithm** | 0.96 | 0.96 | 0.93 | 0.93 | 0.98 | 0.96 | 0.97 | 0.98 | 0.01 | 0.03 | 3.4 | 4.3 |
| **best rater_1_ vs. rater_2_** | 0.98 | 0.98 | 0.95 | 0.96 | 1.00 | 1.00 | 1.00 | 0.99 | 0 | 0 | 1.9 | 2.4 |
| **best rater_1_ vs. algorithm** | 0.98 | 0.97 | 0.96 | 0.95 | 0.99 | 0.98 | 0.99 | 0.98 | 0 | 0 | 1.6 | 2.7 |
| **best rater_2_ vs. algorithm** | 0.97 | 0.98 | 0.95 | 0.95 | 1.00 | 0.98 | 0.99 | 1.00 | 0 | 0 | 2.7 | 3.0 |
| **average rater_1_ vs. rater_2_** | 0.94 | 0.95 | 0.90 | 0.90 | 0.95 | 0.94 | 0.95 | 0.96 | 0.05 | 0.05 | 5.4 | 5.3 |
| **average rater_1_ vs. algorithm** | 0.94 | 0.95 | 0.88 | 0.91 | 0.92 | 0.95 | 0.96 | 0.96 | 0.08 | 0.02 | 7.2 | 5.0 |
| **median rater_2_ vs. algorithm** | 0.95 | 0.94 | 0.90 | 0.90 | 0.95 | 0.93 | 0.95 | 0.96 | 0.05 | 0.06 | 5.1 | 5.5 |
| **SD rater_1_ vs. rater_2_** | 0.02 | 0.02 | 0.04 | 0.04 | 0.04 | 0.04 | 0.04 | 0.03 | 0.06 | 0.04 | 3.0 | 2.8 |
| **SD r_1_ rater_1_ vs. algorithm** | 0.03 | 0.02 | 0.05 | 0.03 | 0.06 | 0.02 | 0.03 | 0.02 | 0.07 | 0.02 | 4.2 | 2.9 |
| **SD rater_2_ vs. algorithm** | 0.02 | 0.02 | 0.03 | 0.04 | 0.04 | 0.05 | 0.03 | 0.03 | 0.04 | 0.06 | 2.5 | 2.7 |
| *Note that sample B was segmented by each rater (number of comparisons = 50), while the respective sample C was evenly split in two subsets for segmentation (number of comparisons = 25, supplementary table S5). The notations “worst” to “best” refer to the performance of the comparisons and correspond to minimum, quartiles and maximum for all columns, except for columns “absolute relative area difference” and “max. Hausdorff distance”, where the order from min to max is descending. The color coding of comparisons corresponds to that of Figure 3 in the main text. SD=one standard deviation.* | | | | | | | | | | | | |

***Supplementary Table S8. Performance evaluation of mitral landmark localization in end-systole on sample B (n=50) and C (n=50)****. The spatial vicinity of the mitral landmarks at the end of the filling phase was within a similar range for the comparisons between humans and algorithm as for the human inter-rater comparison in sample B.*

|  | **sample B (validation subset)** | | | |  | **sample C (test subset)** | | | |
| --- | --- | --- | --- | --- | --- | --- | --- | --- | --- |
|  | **sum of distance deviation (mm)** | | **Absolute angle deviation (in degrees)** | |  | **sum of distance deviation (mm)** | | **Absolute angle deviation (in degrees)** | |
|  | **2ch** | **4ch** | **2ch** | **4ch** |  | **2ch** | **4ch** | **2ch** | **4ch** |
| **median rater_1_ vs. rater_2_** | 4.9 | 5.5 | 2.6 | 2.7 |  |  |  |  |  |
| **median rater_1_ vs. algorithm** | 5.3 | 7.0 | 2.8 | 2.5 |  | 4.9 | 5.3 | 2.8 | 2.7 |
| **median rater_2_ vs. algorithm** | 5.2 | 7.1 | 2.7 | 3.9 |  | 4.3 | 6.3 | 2.5 | 2.7 |
| **worst rater_1_ vs. rater_2_** | 17.0 | 30.1 | 14.0 | 45.5 |  |  |  |  |  |
| **worst rater_1_ vs. algorithm** | 14.0 | 33.4 | 17.1 | 32.6 |  | 10.0 | 10.7 | 9.8 | 11.2 |
| **worst rater_2_ vs. algorithm** | 11.4 | 15.4 | 14.0 | 14.0 |  | 7.6 | 9.0 | 6.6 | 9.0 |
| **worse quartile rater_1_ vs. rater_2_** | 6.4 | 8.1 | 4.2 | 5.1 |  |  |  |  |  |
| **worse quartile rater_1_ vs. algorithm** | 6.9 | 10.4 | 4.6 | 4.7 |  | 5.9 | 7.6 | 4.9 | 4.3 |
| **worse quartile rater_2_ vs. algorithm** | 6.5 | 8.4 | 4.8 | 5.2 |  | 5.2 | 7.2 | 4.1 | 5.1 |
| **better quartile rater_1_ vs. rater_2_** | 3.8 | 4.2 | 0.8 | 1.2 |  |  |  |  |  |
| **better quartile rater_1_ vs. algorithm** | 3.7 | 5.7 | 1.8 | 1.3 |  | 3.4 | 4.5 | 1.7 | 2.1 |
| **better quartile rater_2_ vs. algorithm** | 3.6 | 4.7 | 1.3 | 1.1 |  | 3.0 | 5.3 | 0.9 | 1.3 |
| **best rater_1_ vs. rater_2_** | 3.0 | 1.3 | 0 | 0 |  |  |  |  |  |
| **best rater_1_ vs. algorithm** | 0 | 2.3 | 0 | 0 |  | 1.6 | 1.6 | 0 | 0 |
| **best rater_2_ vs. algorithm** | 0 | 1.3 | 0 | 0.3 |  | 1.3 | 3.0 | 0 | 0 |
| **average rater_1_ vs. rater_2_** | 5.7 | 6.7 | 3.1 | 4.5 |  |  |  |  |  |
| **average rater_1_ vs. algorithm** | 5.6 | 8.9 | 3.5 | 4.2 |  | 5.0 | 5.7 | 3.2 | 3.6 |
| **average rater_2_ vs. algorithm** | 5.4 | 6.9 | 3.8 | 3.7 |  | 4.3 | 6.2 | 2.6 | 3.3 |
| **SD rater_1_ vs. rater_2_** | 2.8 | 5.2 | 2.8 | 7.4 |  |  |  |  |  |
| **SD r_1_ rater_1_ vs. algorithm** | 3.1 | 5.5 | 3.0 | 5.7 |  | 2.2 | 2.3 | 2.4 | 2.7 |
| **SD rater_2_ vs. algorithm** | 2.4 | 3.3 | 3.6 | 3.4 |  | 1.5 | 1.6 | 2.1 | 2.8 |
| *For 2ch the hinge points are inferior and anterior, while for 4ch lateral and septal. Note that sample C (N=50) was evenly split for segmentation by the two raters on the end-systole frame, while the sample B was segmented by each rater. The notations “worst” to “best” refer to the performance of the comparisons and correspond to minimum, quartiles and maximum for all columns, except for columns “absolute relative area difference” and “max. Hausdorff distance”, where the order from min to max is descending. SD = one standard deviation.* | | | | | | | | | |

***Supplementary Table S9. Performance evaluation of longitudinal diameters at end-systole in sample C.*** *Descriptive statistics of longitudinal diameters at end-systole of LAx cines (absolute values in cm) in sample C, geometrically reconstructed from the manual contours of the left atrium and the mitral landmarks based on the respective left atrial segmentation mask and mitral annular hinge points with two methods: “center of mass”, as the line starting at the mid-point of the mitral annular level (line connecting the two respective mitral landmarks) and passing through the center of mass of the segmentation of the left atrium and “perpendicular”, as the longest perpendicular line from the mitral annular level to the posterior wall of the atrium.*

| **diameter calculation method** |  | **center of mass** | |  | **perpendicular** | |
| --- | --- | --- | --- | --- | --- | --- |
|  |  | **2ch** | **4ch** |  | **2ch** | **4ch** |
| **Median** |  | 5.0 | 5.9 |  | 5.1 | 5.7 |
| **Minimum** |  | 3.2 | 3.8 |  | 3.1 | 3.8 |
| **1^st^ quartile** |  | 4.7 | 5.3 |  | 4.3 | 4.9 |
| **3^rd^ quartile** |  | 5.6 | 6.5 |  | 5.7 | 6.3 |
| **Maximum** |  | 8.2 | 7.8 |  | 8.5 | 7.7 |
| **Average** |  | 5.2 | 5.9 |  | 5.1 | 5.7 |
| **one standard deviation** |  | 0.9 | 0.9 |  | 1.0 | 0.9 |

**Supplementary Table S10. Summary of Bland-Altman analysis performed for the absolute maximal left atrial volumes and left atrial volumes index for LAx cines.** Following comparisons were performed for both variants of longitudinal diameter calculation: i) in sample B for the inter-rater comparison; ii) in sample C for the comparison between volumes calculated from human segmentations (subscript h) and volumes calculated from automated segmentations on human selected ES frame (subscript a); iii) in sample C for the comparison between volumes calculated from human segmentations and fully automated volume calculation (subscript Atri-U). A positive sign denotes an underestimation, while a negative sign an overestimation, compared to the first value set. There is a similar average bias between the raters in the validation sample (C: ${Vol}_{max}^{rater1}$ - ${Vol}_{max}^{rater2}$) and between human and fully automated analysis in the test sample (C: ${Vol}_{max}^{h}$ - ${Vol}_{max}^{Atri-U}$), with a maximal average overestimation of 1.6 mL for the “center of mass" method of diameter calculation. For this comparison, especially the lower limit of agreement (1.96 standard deviation) is higher than the inter-rater comparison.

|  |  | **LAV_max_ (mL)** | |  | **LAVi_max_ (mL/m^2^)** | |
| --- | --- | --- | --- | --- | --- | --- |
|  |  | **Bias** | **Limits of agreement, lower to upper** |  | **Bias** | **Limits of agreement, lower to upper** |
| **Validation sample (N=50)** |  |  |  |  |  |  |
| C: ${Vol}_{max}^{rater1}$ - ${Vol}_{max}^{rater2}$ |  | -0.3 | -13.5 to 13.0 |  | -0.2 | -7.0 to 6.6 |
| P: ${Vol}_{max}^{rater1}$ - ${Vol}_{max}^{rater2}$ |  | -1.9 | -16.4 to 12.5 |  | -1.0 | -8.4 to 6.3 |
| **Test sample (N=50)** |  |  |  |  |  |  |
| C: ${Vol}_{max}^{h}$ - ${Vol}_{max}^{a}$ |  | 1.0 | -9.8 to 11.8 |  | 0.6 | -4.9 to 6.0 |
| C: ${Vol}_{max}^{h}$ - ${Vol}_{max}^{Atri-U}$ |  | -1.6 | -15.1 to 11.9 |  | -0.8 | -7.5 to 5.8 |
| P: ${Vol}_{max}^{h}$ - ${Vol}_{max}^{a}$ |  | 1.7 | -11.0 to 14.5 |  | 0.9 | -5.5 to 7.4 |
| P: ${Vol}_{max}^{h}$ - ${Vol}_{max}^{Atri-U}$ |  | -1.4 | -15.0 to 12.2 |  | -0.7 | -7.7 to 6.2 |
| *Bias: mean difference between volumes, limits of agreement: two standard deviations of differences. Abbreviations: h: human, a: algorithm. C: center of mass method, P: perpendicular method of longitudinal diameter calculation, LAV: left atrial volume, LAVi: left atrial volume index.* | | | | | | |

**Supplementary Figure S5. Bland-Altman analyses for the estimated left atrial volume index** (${LAVi}_{max}$, in mL/m^2^) with the two longitudinal axis calculation methods. Upper row: based on center of mass method. Lower row: based on the perpendicular method. Left column: human versus automated segmentations on the visually selected end-systolic time-frame. Right column: human versus fully automated calculation (Atri-U). Each window contains 50 values. The dashed line in the middle of the graph represents the bias estimate, while the upper and lower dashed lines the upper and lower limits of agreement (two standard deviations of bias), respectively. The color shadings represent the respective 95% confidence intervals of the bias estimate (blue) and the lower and upper limits of agreement (red and green, respectively).

|  | Indexed left atrial volumes from human vs. automated segmentations on the visually selected end-systolic time-frame |  | Indexed left atrial volumes from human segmentations vs. Atri-U |
| --- | --- | --- | --- |
| Center of mass method | 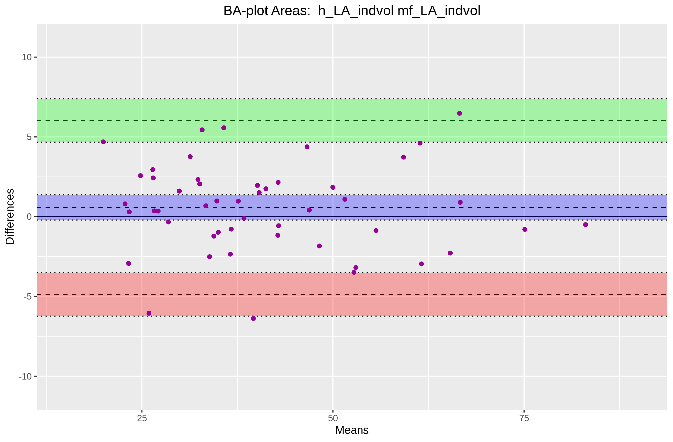 |  | 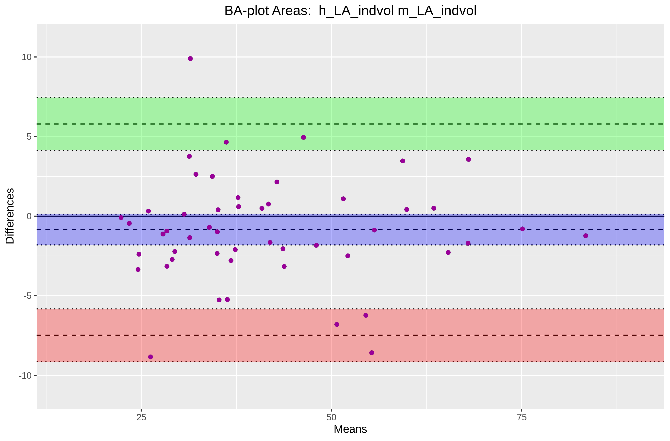 |
| Perpendicular method | 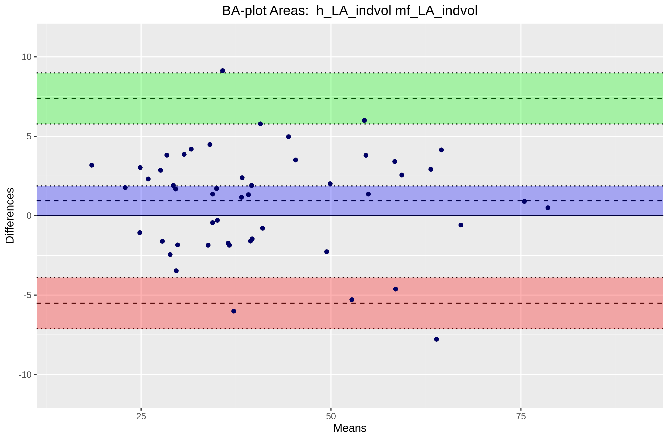 |  | 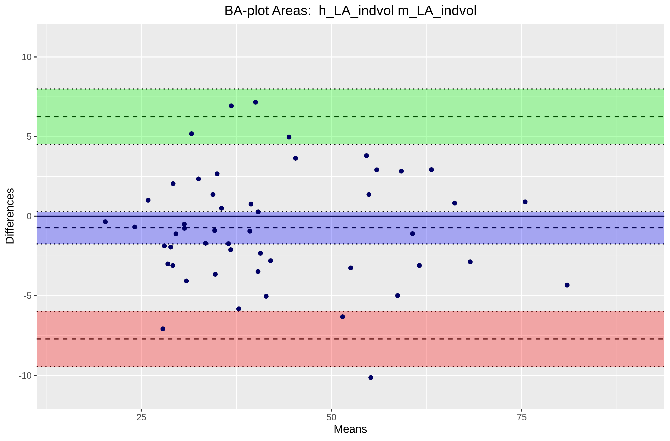 |

**Supplementary Figure S6 (as an extension to Figure 4 in the main document). Distributions of the maximum indexed left atrial volumes (LAVi_max_) in sample C.** The two halves represent the volume estimation with the two different variants of longitudinal atrial calculation. The left half represents indexed volumes estimated from the perpendicular method, while the right half represents indexed volumes estimated from the center of mass method. The left violin plot represents values from human segmentations, the middle plot values from automated segmentations on the end-systolic time-frame selected by the human rater, while the right plot represents values from the fully automated solution (Atri-U), respectively. In general, the lower and upper boundaries of the violin plot represent minimum and maximum values, respectively, while the black bar in the center is the interquartile range (first and third quartile, from bottom to top respectively). The bright dot in the middle is the median value. The distribution of the underlying data (scatter) is represented by the curved sides of the plot.


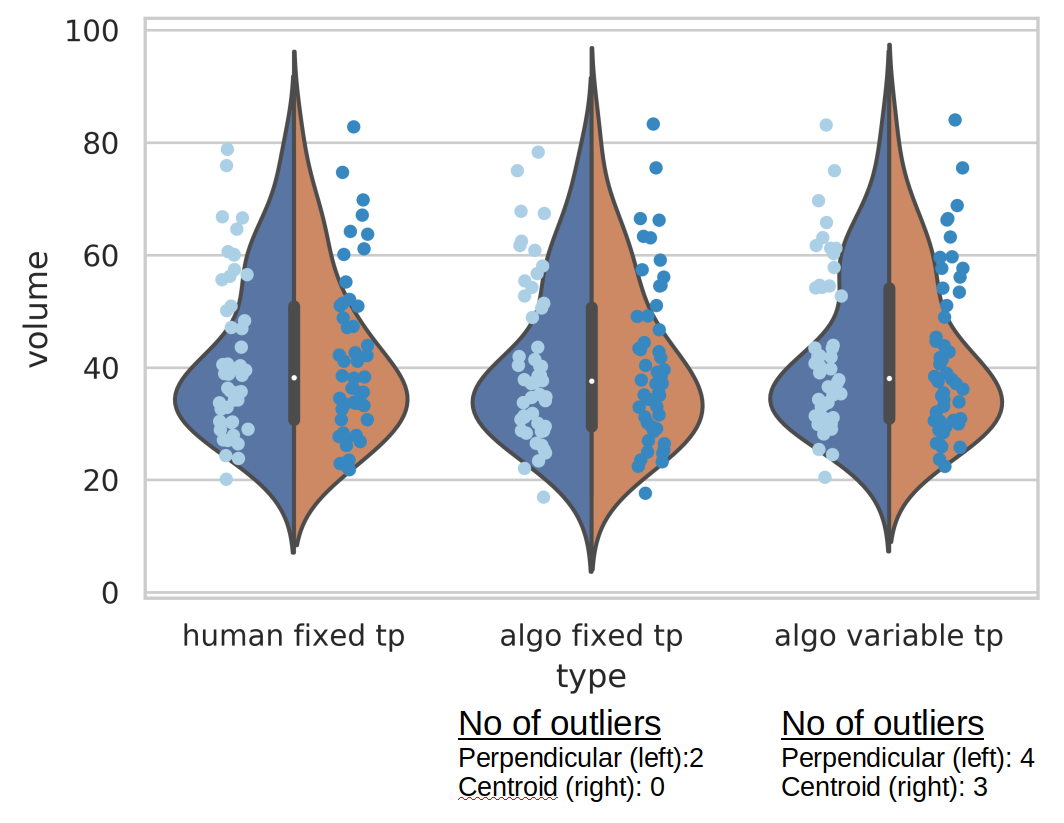


# Section E2

## Manual annotation

The datasets were processed after having discussed discrepancies in preliminary datasets. Manual annotation was performed with a mouse-driven cursor in the image-analysis software Nora ([www.nora-imaging.com](http://www.nora-imaging.com)). The manual processing consisted of three partially interdependent steps. In step 1, the mitral valve state (open/closed) was specified in every frame of the 4ch-views. In step 2, the endocardial borders of the left atrium were segmented on the frame directly prior to the opening of the mitral valve, corresponding to ventricular end-systole (ES), for both the 2ch- and 4ch-view, excluding the left atrial appendage and pulmonary vein orifices (contrary to the inclusion of the left atrial appendage as described in *Hudsmith LE, Petersen SE, Francis JM, Robson MD, Neubauer S. Normal human left and right ventricular and left atrial dimensions using steady state free precession magnetic resonance imaging. J Cardiovasc Magn Reson 2005; 7(5):775–82*). In step 3, mitral annular hinge point pairs were segmented in the 2ch- (inferior and anterior) and 4ch-view (lateral and septal), respectively (referred as mitral landmarks throughout the text). In samples A and B the mitral landmarks were additionally segmented at ventricular end-diastole and at a third, randomly predefined frame among the remaining frames. The processing, involving steps 1 to 3 and additionally the segmentation of the right atrium (not further analyzed within the scope of the present study), was timed for each rater in sample B.

Processing of the cines was randomly and equally assigned to one of the raters (T.A.D or C.A.) for samples A and C and to both raters for sample B. They were blinded to clinical information. They required between 100 and 240 seconds (average 185 seconds) and processing times for the separate steps were estimated as follows: 1x5 seconds for the ES frame selection, 3x40 seconds for atrial segmentation and 6 x 10 seconds for mitral landmark or diameter placement.

## Deep learning algorithms and post-processing methods

The manual segmentations of both raters were used for the training. The two-dimensional LAx data was processed with two-dimensional convolutions and four resolution levels, resulting in a spatial context of (89x89). This means that the prediction at a voxel depended on the neighborhood of 44 in-plane voxels around that voxel. We used the same number of feature channels and pooling/up-convolution layers as in (*Çiçek Ö, Abdulkadir A, Lienkamp SS, Brox T, Ronneberger O. 3D U-Net: Learning Dense Volumetric Segmentation from Sparse Annotation. In: Proceedings of MICCAI 2016:424–32.17*) and in (*Ronneberger O, Fischer P, Brox T. U-Net: Convolutional Networks for Biomedical Image Segmentation. In: Proceedings of MICCAI 2015:234–41*). The manual segmentations of both raters were used as ground truth for the training. The patches for training were randomly sampled from the training data such that the likelihood that the central voxel of the patch had equal probability of being background or foreground. To reduce the risk of numerical instability because of large gradients, the gradient norm was clipped to 0.5. Training was performed with stochastic gradient descent with batch size of eight, learning rate of 0.01 and momentum 0.9 and trained for 50,000 iterations with halving the learning rate every 10,000 iterations. A simple post-processing step on the left atrium segmentations followed, during which only the largest area connected component was kept, ensuring that spurious remote segmentations were excluded beforehand.

The detection of the mitral landmarks (mitral annular hinge points) was also solved as a segmentation task. After smoothing the ground truth landmarks with a 3mm wide Gaussian kernel, they were used as dense labels. The landmark position was then predicted on all frames as the position of the local maximum of the predicted probability of each mitral landmark. The less probable localizations over all frames were omitted, in order to achieve a maximum of two localizations. Finally, the coordinates of localizations were smoothed over all frames with a median temporal filter and a window size of 5, in order to correct missing or spurious predicted coordinates.

In conclusion, for the segmentation algorithm of the left atrium area in 2ch- and 4ch-views, the available CMR images were N=200, 50 and 50 for the training, validation and test subset (sample A, B and C, respectively). For the mitral landmark algorithm, the available images were N=600, 150 and 50, respectively (as the landmarks were segmented in 200 cases and 3 time-frames in samples A and B, while sample C was segmented only in the ES phase).

The mitral valve states were classified on each 4ch-cine frame using a fine-tuned deep fully convolutional neural network with 18 residual layers [9]. The first two layers of ResNet are the 7×7 convolutional layer with 64 output channels and a stride of 2, followed by a 3×3 maximum pooling layer with a stride of 2. A batch normalization layer is added after each convolutional. After 4 residual blocks (each two layers deep) with input sizes (64, 128, 256, 512) a final average pooling with a softmax is added at the end. The network was optimized with Stochastic Gradient Descent with an initial learning rate of 0.001 and a momentum of 0.9. The learning rate was reduced every 7 epochs by 0.1. For the frame classification algorithm there were N=5000, 1,250 and 1,250, for training, validation, and test (A, B and C respectively). The prediction of the classification algorithm was undertaken on each frame and was labelled as 1 when the valve was closed and as 0 when the valve was open. Inconsistent individual mitral valve states were corrected with a morphological one-dimensional closing operation (i.e. if the states of the time points before and after were different, the state was changed to the state that the neighbors). After this post-processing step, if the predicted states formed more than one block of closed states the last frame of the longest block was picked as the end-systolic time point, whereas if the blocks were equally long, the last frame of the second (later in time) block was chosen.

## Computation of left atrial volume from the 2D cines

Finally, the ${LAV}_{max}$ was computed using the biplane LAx area‐length method (in mL):

$${LAV}_{max}=\frac{8}{3\pi}\frac{A_{2ch}{\cdot A}_{4ch}}{L}$$

where $A_{2ch}$ and $A_{4ch}$ (in cm^2^) refer to the area of the left atrium at ES in 2ch- and 4ch-views, respectively and $L$ to the length of the shorter of the two longitudinal atrial diameters (in cm).

**Supplementary Figure S7. Susceptibility of left atrial volume to time frame selection.** For sample C (N=50), the left atrial volume (LAV_t_, in ml) was computed for all 25 time points from automatically predicted left atrial segmentations and diameters. The percentage delta to the LAV_max_ was calculated per case as follows Delta_perc_ = LAV_t_/LAV_max_ and boxplots were plotted for all timeframes. Note that the median values (line inside the boxplot) follows the expected left atrial volume curve (starting from the minimal volume) with: a slow increase until ventricular ES, then a steep decrease to the plateau preceding atrial contraction (approximately at ES + 8), before returning to the minimal volume. Note also the larger 1^st^ and 3^rd^ quartiles (lower and upper margin of the boxplot, respectively) in the frames immediately after the opening of the mitral valve (ES+1 to ES+5) compared to the five frames preceding the opening of the mitral valve (ES-5 to ES-1). This might have physiological causes, but could also be due to the ill-defined borders of the atrium and the consecutive erroneous segmentations when the mitral valve is open.


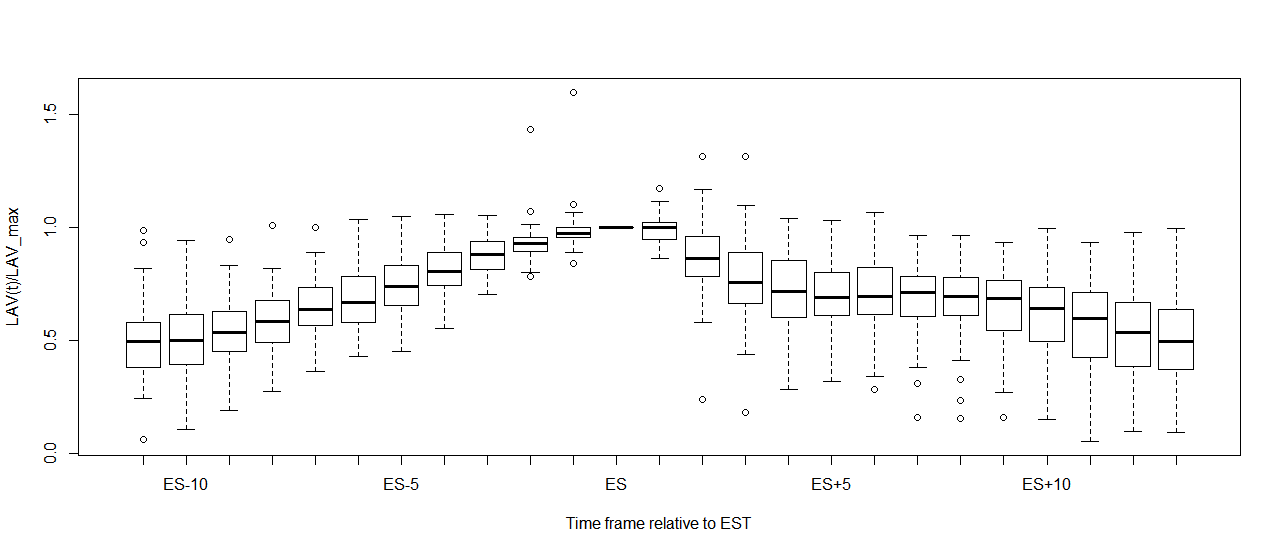


# Section E3 - 3D cines

## Dataset

Axial stacks of multislice b-SSFP cines (3D cines) covering the left atrium originated from a protocol dedicated to the preablation planning of atrial fibrillation and were acquired between 2010 and 2014 from 187 patients at 1.5 Tesla (Magnetom Espree, Siemens, Erlangen, Germany). For the purpose of this study, 6 out of 187 cases were excluded due to low image quality. From the remaining cases, 65 cases were randomly selected and assigned into batches for training ($N=50$), validation ($N=5$) and testing ($N=10$) of a neural network model for semantic segmentation of the left atrium.

The cardiac cycle was sampled with a median of 25 time-frames (range: 14 to 26) with retrospective ECG gating. Acquisition matrix was 156x192 (N=64) or 174x192 (N=1) and pixel size was 1.46x1.46 to 2.1x2.1 mm^2^. The slice thickness was 6 mm (N=64) or 6.6 mm (N=1) and the number of slices 6 to 13 with no interslice gap. The size of the left atrium spanned 7 to 13 axial slices in the 3D cine dataset. Demographic and clinical characteristics are shown in **Supplementary Table S-3D(a)**. Written informed consent was obtained before inclusion in the study (ClinicalTrials.gov Identifier: NCT03718364).

**Supplementary Table S-3D(a). Basic demographic and clinical characteristics of the 3D cine population**

|  |  | **Training** |  | **Validation** |  | **Test** |  |
| --- | --- | --- | --- | --- | --- | --- | --- |
| **Number of cases** |  | **50** |  | **5** |  | **10** |  |
| **Age in years**;  median (range) [q1, q3] |  | 65 (33–79)  [56, 70] |  | 67 (62–71)  [64, 70] |  | 64 (56–69)  [63, 66] |  |
| **Sex**; male/female |  | 34 / 16 |  | 4 / 1 |  | 10 / 0 |  |
| **Body surface area in m^2^**; median (range) [q1, q3] |  | 1.95 (1.50–2.57)  [1.73, 2.09] |  | 2.10 (1.88–2.46)  [2.07, 2.28] |  | 2.04 (1.76–2.29)  [1.96, 2.09] |  |
| **Atrial fibrillation type** |  |  |  |  |  |  |  |
| - paroxysmal |  | 28 |  | 3 |  | 9 |  |
| - persistent |  | 22 |  | 2 |  | 1 |  |
| **Left atrial dilatation** |  | 27 |  | 3 |  | 5 |  |
| *Abbreviations: q1/q3: 1^st^/3^rd^ quartile* | | | | | | | |

## Manual contouring

For 3D cines, manual contouring of the endocardial borders of the left atrium was performed for each axial slice in all frames of the training and validation subsets by two raters (M.P. and S.K.), with the help of a semi-automated algorithm propagating to neighboring frames in the software Segment (version 2.2 R6435, <http://segment.heiberg.se>). The mitral annular level served as the border to the left ventricle, while the left atrial appendage and pulmonary veins were excluded at their orifices. For the test subset, segmentation was performed in the end-systolic (ES) frame, the end-diastolic (ED) frame and the frame preceding the onset of atrial contraction (preA), which were selected visually.

## Automated segmentation

A 3D U-Net architecture was used with the same number of feature channels as for the LAx dataset (see above). To integrate the entire spatial information contained in the image stacks forming the 3D datasets while accounting for the anisotropic resolution, we mixed two- and three-dimensional unpadded convolutions. Specifically, the last resolution level consisted of convolutions in three spatial dimensions (3x3x3 convolution kernels and 2x2x2 pooling/up-convolution kernels), whereas all the operations in higher resolutions were only in-plane (3x3x1 convolution kernels and 2x2x1 pooling/up-convolution kernels). This resulted in a spatial context of 89x89x5. Thus, the prediction at a voxel depended on the neighborhood of 44 in-plane voxels around that voxel and two slices above/below.

The comparisons between human raters and algorithms were performed at all time-frames in the validation subset and for the frames ED, ES and preA in the test subset. Median Dice coefficients between manually defined and automated left atrial segmentations in the test subset were 0.92 with rater_1_ and 0.94 with rater_2_ at the ES phase, 0.89 with rater_1_ and 0.90 with rater_2_ at the ED phase, and 0.90 with rater_1_ and 0.93 with rater_2_ at the preA phase. The detailed comparison results are presented in **Supplementary Table S-3D(b)**.

**Supplementary Table S-3D(b). Comparison metrics for the left atrium volume in the 3D validation (N=5) and test (N=10) subsets*** Performance evaluation metrics for the validation set and comparisons between the two human raters, rater_1_ vs. Atri-U and rater_2_ vs. Atri-U. Overlap of segmentation between human and 3D Atri-U in the test set was similar to the inter-rater overlap in the validation set. For the test set the metrics were calculated separately for the distinct phases of left atrium (ES, ED and preA) and similar segmentation overlaps were achieved.

|  | **Validation subset** | | | | | |  | **Test subset** | | | | | | | | | | | | | | | | | |
| --- | --- | --- | --- | --- | --- | --- | --- | --- | --- | --- | --- | --- | --- | --- | --- | --- | --- | --- | --- | --- | --- | --- | --- | --- | --- |
|  | **Dice coefficient** | **Jaccard index** | **Pixelwise precision** | **Pixelwise recall** | **Absolute relative volume difference** | **Max. Hausdorff distance (mm)** |  | **Dice coefficient** | | | **Jaccard index** | | | **Pixelwise precision** | | | **Pixelwise recall** | | | **Absolute relative volume difference** | | | **Max. Hausdorff distance (mm)** | | |
| **Time points** | **all** | **all** | **all** | **all** | **all** | **all** |  | **ES** | **ED** | **preA** | **ES** | **ED** | **preA** | **ES** | **ED** | **preA** | **ES** | **ED** | **preA** | **ES** | **ED** | **preA** | **ES** | **ED** | **preA** |
| **median rater_1_ vs. rater_2_** | 0.92 | 0.84 | 0.88 | 0.95 | 0.09 | 9.8 |  |  |  |  |  |  |  |  |  |  |  |  |  |  |  |  |  |  |  |
| **median rater_1_ vs. algorithm** | 0.92 | 0.85 | 0.88 | 0.95 | 0.09 | 10.9 |  | 0.92 | 0.89 | 0.90 | 0.85 | 0.81 | 0.82 | 0.88 | 0.83 | 0.84 | 0.98 | 0.98 | 0.99 | 0.11 | 0.15 | 0.13 | 11.9 | 8.0 | 12.2 |
| **median rater_2_ vs. algorithm** | 0.92 | 0.86 | 0.92 | 0.93 | 0.02 | 8.4 |  | 0.94 | 0.90 | 0.93 | 0.89 | 0.81 | 0.87 | 0.93 | 0.93 | 0.94 | 0.95 | 0.87 | 0.95 | 0.04 | 0.12 | 0.10 | 6.3 | 10.1 | 8.0 |
| **worst rater_1_ vs. rater_2_** | 0.85 | 0.74 | 0.78 | 0.88 | 0.20 | 13.4 |  |  |  |  |  |  |  |  |  |  |  |  |  |  |  |  |  |  |  |
| **worst rater_1_ vs. algorithm** | 0.88 | 0.79 | 0.82 | 0.90 | 0.19 | 15.5 |  | 0.91 | 0.84 | 0.89 | 0.83 | 0.73 | 0.80 | 0.84 | 0.73 | 0.80 | 0.93 | 0.86 | 0.91 | 0.16 | 0.27 | 0.19 | 12.6 | 12.5 | 14.6 |
| **worst rater_2_ vs. algorithm** | 0.89 | 0.81 | 0.87 | 0.86 | 0.11 | 12.6 |  | 0.92 | 0.84 | 0.89 | 0.86 | 0.73 | 0.80 | 0.91 | 0.85 | 0.87 | 0.87 | 0.79 | 0.82 | 0.12 | 0.15 | 0.19 | 9.0 | 12.0 | 10.9 |
| **worse quartile rater_1_ vs. rater_2_** | 0.90 | 0.82 | 0.85 | 0.92 | 0.13 | 11.1 |  |  |  |  |  |  |  |  |  |  |  |  |  |  |  |  |  |  |  |
| **worse quartile rater_1_ vs. algorithm** | 0.91 | 0.83 | 0.86 | 0.94 | 0.12 | 12.5 |  | 0.92 | 0.87 | 0.89 | 0.84 | 0.77 | 0.81 | 0.86 | 0.81 | 0.82 | 0.97 | 0.96 | 0.95 | 0.12 | 0.19 | 0.18 | 12.5 | 11.7 | 13.3 |
| **worse quartile rater_2_ vs. algorithm** | 0.91 | 0.84 | 0.91 | 0.91 | 0.06 | 9.4 |  | 0.93 | 0.89 | 0.92 | 0.88 | 0.80 | 0.85 | 0.92 | 0.91 | 0.94 | 0.94 | 0.83 | 0.89 | 0.06 | 0.15 | 0.10 | 7.3 | 11.1 | 10.1 |
| **better quartile rater_1_ vs. rater_2_** | 0.93 | 0.86 | 0.94 | 0.97 | 0.05 | 8.2 |  |  |  |  |  |  |  |  |  |  |  |  |  |  |  |  |  |  |  |
| **better quartile rater_1_ vs. algorithm** | 0.93 | 0.86 | 0.92 | 0.97 | 0.03 | 7.7 |  | 0.93 | 0.90 | 0.91 | 0.86 | 0.82 | 0.84 | 0.91 | 0.85 | 0.86 | 0.98 | 0.99 | 0.99 | 0.05 | 0.11 | 0.11 | 7.3 | 8.0 | 8.4 |
| **better quartile rater_2_ vs. algorithm** | 0.93 | 0.87 | 0.93 | 0.95 | 0.01 | 7.5 |  | 0.94 | 0.91 | 0.95 | 0.89 | 0.83 | 0.90 | 0.97 | 0.93 | 0.97 | 0.96 | 0.97 | 0.96 | 0.03 | 0.06 | 0.02 | 6.0 | 8.2 | 7.1 |
| **best rater_1_ vs. rater_2_** | 0.96 | 0.92 | 0.98 | 0.99 | 0.01 | 6.0 |  |  |  |  |  |  |  |  |  |  |  |  |  |  |  |  |  |  |  |
| **best rater_1_ vs. algorithm** | 0.94 | 0.89 | 0.95 | 0.98 | 0 | 6.0 |  | 0.94 | 0.90 | 0.92 | 0.89 | 0.82 | 0.85 | 0.92 | 0.88 | 0.92 | 0.99 | 1 | 0.99 | 0.03 | 0.02 | 0.01 | 6.3 | 7.3 | 6.8 |
| **best rater_2_ vs. algorithm** | 0.95 | 0.90 | 0.95 | 0.96 | 0 | 6.0 |  | 0.95 | 0.95 | 0.95 | 0.91 | 0.91 | 0.90 | 0.98 | 0.95 | 0.97 | 0.97 | 0.97 | 0.98 | 0.03 | 0.04 | 0.01 | 6.0 | 6.6 | 6.0 |
| **average rater_1_ vs. rater_2_** | 0.91 | 0.84 | 0.89 | 0.94 | 0.09 | 9.7 |  |  |  |  |  |  |  |  |  |  |  |  |  |  |  |  |  |  |  |
| **average rater_1_ vs. algorithm** | 0.91 | 0.84 | 0.88 | 0.95 | 0.08 | 10.5 |  | 0.92 | 0.88 | 0.90 | 0.86 | 0.79 | 0.82 | 0.88 | 0.82 | 0.85 | 0.97 | 0.96 | 0.97 | 0.09 | 0.15 | 0.13 | 10.1 | 9.5 | 11.0 |
| **average rater_2_ vs. algorithm** | 0.92 | 0.86 | 0.92 | 0.93 | 0.03 | 8.6 |  | 0.94 | 0.90 | 0.93 | 0.89 | 0.82 | 0.86 | 0.94 | 0.92 | 0.94 | 0.94 | 0.89 | 0.92 | 0.06 | 0.10 | 0.08 | 6.9 | 9.6 | 8.4 |
| **SD rater_1_ vs. rater_2_** | 0.02 | 0.04 | 0.05 | 0.03 | 0.05 | 1.8 |  |  |  |  |  |  |  |  |  |  |  |  |  |  |  |  |  |  |  |
| **SD r_1_ rater_1_ vs. algorithm** | 0.01 | 0.03 | 0.03 | 0.02 | 0.05 | 2.7 |  | 0.01 | 0.02 | 0.01 | 0.02 | 0.04 | 0.02 | 0.03 | 0.06 | 0.05 | 0.02 | 0.06 | 0.04 | 0.05 | 0.09 | 0.07 | 3.0 | 2.4 | 3.3 |
| **SD rater_2_ vs. algorithm** | 0.01 | 0.02 | 0.02 | 0.02 | 0.03 | 1.6 |  | 0.01 | 0.04 | 0.02 | 0.02 | 0.06 | 0.04 | 0.03 | 0.04 | 0.04 | 0.04 | 0.08 | 0.06 | 0.04 | 0.05 | 0.07 | 1.3 | 2.2 | 2.1 |
| **note that the validation subset was segmented by each rater, while the test subset was evenly split in two subsets for segmentation.*  *Abbreviations: ES: end-systole, ED: end-diastole, preA: directly preceding atrial contraction. SD=one standard deviation.*  *The notations “worst” to “best” refer to the performance of the comparisons and correspond to minimum, quartiles and maximum for all columns, except for columns “absolute relative area difference” and” max. Hausdorff distance”, where the order from min to max is descending.* | | | | | | | | | | | | | | | | | | | | | | | | | |

## Computed left atrial volume from the 3D cines

The left atrial volume at each frame (${LAV}_{frame}^{3D}$) was calculated from the equation (in mL):

$${LAV}_{frame}^{3D}=\sum_{s=1}^{n} A_{s}\cdot t$$

multiplying the chamber area $A_{S}$ (in cm^2^) on each slice s, times the slice thickness t (in cm) and summing these products over all slices 1,…,n. Results are shown in **Supplementary** **Table** **3D(c)** and **Supplementary Figure** **S-3D(a)**.

**Supplementary Table S-3D(c).** Descriptive statistics of maximal left atrial volume (from rater_1_).

| **subsets** |  | **Training†** |  | **Validation†** |  | **Test*** |
| --- | --- | --- | --- | --- | --- | --- |
| **Subjects (N)** |  | 50 |  | 5 |  | 10 |
| **median** |  | 105.1 |  | 114.8 |  | 96.1 |
| **SD** |  | 38.5 |  | 17.3 |  | 21.8 |
| **minimum** |  | 55.8 |  | 93.5 |  | 51.2 |
| **1^st^ quartile** |  | 82.2 |  | 105.4 |  | 87.8 |
| **3^rd^ quartile** |  | 123.3 |  | 131.0 |  | 112.3 |
| **maximum** |  | 264.3 |  | 135.0 |  | 120.6 |
| **average** |  | 110.3 |  | 115.9 |  | 94.6 |
| **SD** |  | 38.5 |  | 17.3 |  | 21.8 |
| ** at ES frame, † at frame with maximal volume. SD=one standard deviation.* | | | | | | |

**Supplementary Figure S-3D(a)**. **Indexed** **volume of a 3D cine in all recorded 25 time-frames**, in a case with paroxysmal atrial fibrillation. Left: the blue dots represent index volume values from automated calculation, while the blue circles the segmentation by human raters at the three distinct phases (ES, ED, preA). Right: a side-by-side comparison of segmentations. In blue is automated segmentation and red is human segmentation. See also Video 2 for the segmentation results at all three phases and all slices.


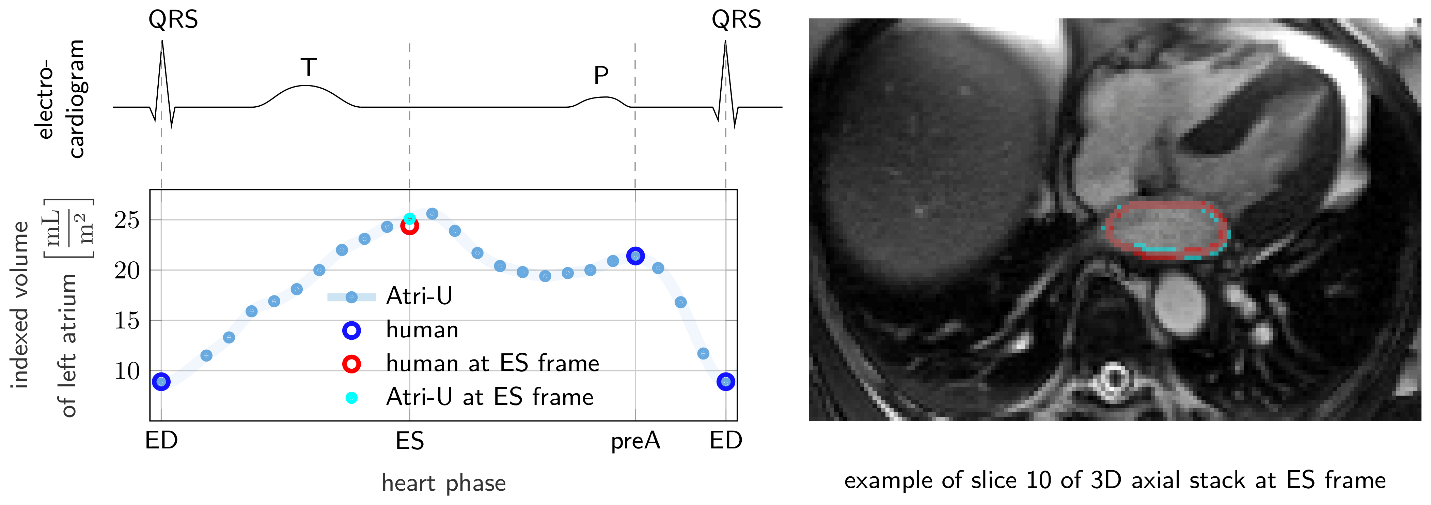


Bland-Altman analyses between human rater and algorithm segmentations were performed for the indexed left atrial volumes ${LAVi}_{frame}^{3D}$ (in mL/m^2^). The average underestimation, over frames ED, ES and preA, for the indexed volume was 2.4 mL/m^2^, slightly lower than the average inter-rater bias of 3.9 mL/m^2^ (see **Supplementary Table S-3D(d)**), yet with broader limits of agreement. Overall, the predictions were accurate over the different phases of the cardiac cycle (see **Supplementary Figure S-3D(b)**).

**Supplementary Table S-3D(d). Summary of Bland-Altman analysis for 3D cines over all phases of the cardiac cycle**: inter-rater comparison in the validation set and comparison between human (subscript h, combining data from both raters) and automated segmentation (subscript a) in the test set. Compared to the first value set, a positive and a negative sign denote an under- or overestimation, respectively.

|  |  | **LAV_max_ (mL)** | |  | **LAVi_max_ (mL/m^2^)** | |
| --- | --- | --- | --- | --- | --- | --- |
| **Subset** | **Volume_A_-Volume_B_** | **Bias** | **Limits of agreement, lower to upper** |  | **Bias** | **Limits of agreement, lower to upper** |
| **Validation (n=5x25=125)** | ${Vol}^{rater1}$ - ${Vol}^{rater2}$ | 8.4 | 0.9 to 15.9 |  | 3.9 | 0.4 to 7.3 |
| **Test (n=10x3=30)** | ${Vol}^{human}$- ${Vol}^{Atri-U}$ | 4.6 | -12.1 to 21.3 |  | 2.4 | -6.3 to 11.1 |
| *Bias: mean difference between volumes, limits of agreement: two standard deviations of differences. Abbreviations: LAV: left atrial volume, LAVi: left atrial volume index* | | | | | | |

**Supplementary Figure S-3D(b). Bland-Altman analyses for the indexed left atrial volume from 3D cines.** *Bland-Altman plots of indexed left atrium 3D volumes at three distinct phases of the cardiac cycle for the 10 subjects of the test set (in mL/m^2^, 30 values). Each triplet with the same color belongs to one subject, while each phase of the cardiac cycle is depicted with different marker shapes (circle: end-diastole, triangle: end-systole and rectangle: directly preceding atrial contraction). The automated calculation shows an average bias of 2.4mL/m^2^.*


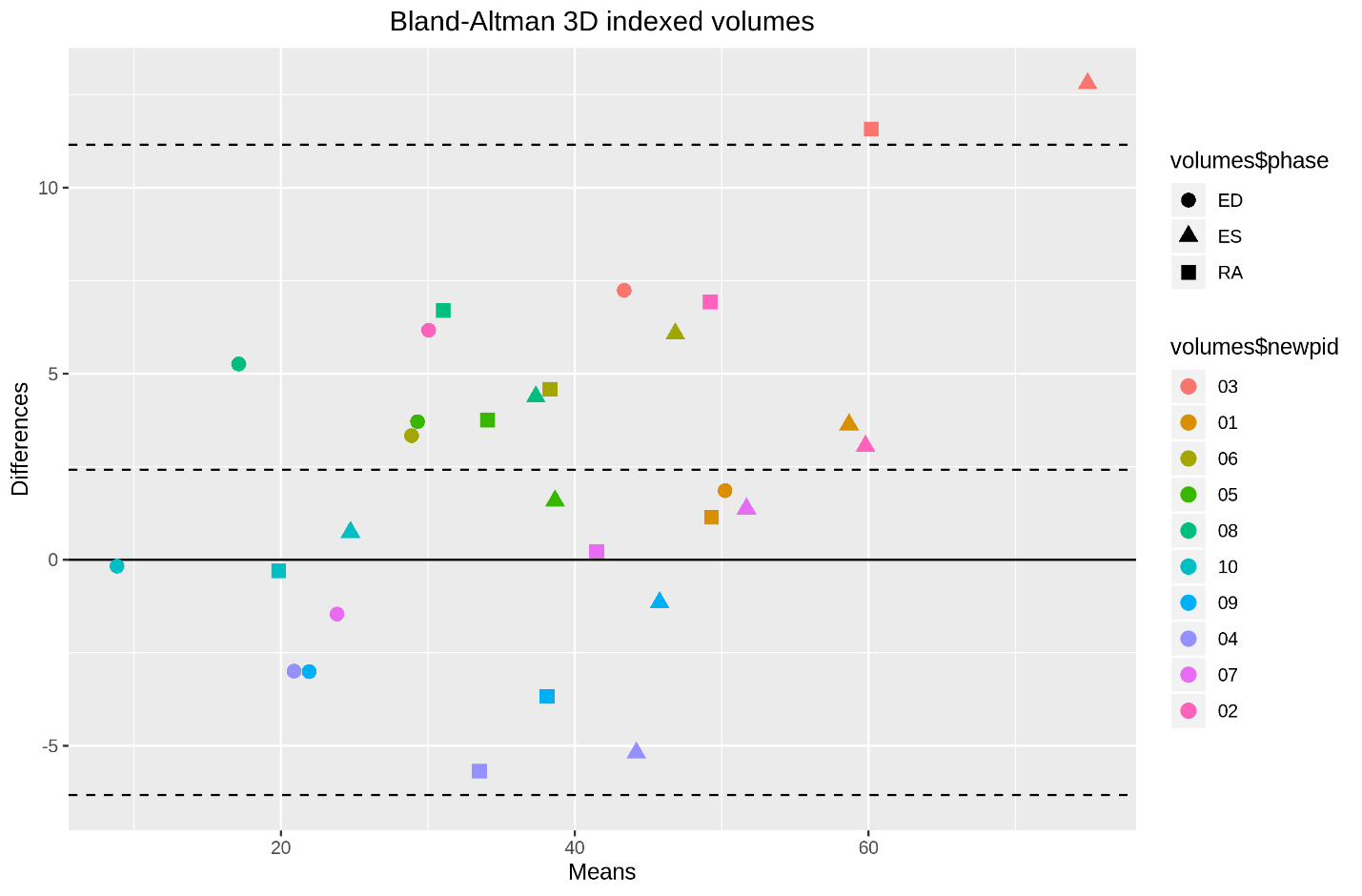


# Section E4

## Dataset

The hospital information system of our institution was searched for additional examinations with coexisting biplane long-axis and transversal multistack cines from patients that accepted the general consent that their data may be used for research. Twenty-two studies fulfilling these criteria were found, acquired between April 2017 and January 2021. Four studies were excluded applying the same criteria as for algorithm development: 1 with an atrial tumor, 2 with a tetralogy of Fallot, and 1 with mitral valve sclerosis, while one additional study was excluded as images of the patient had been used in the 2D algorithm training. The 17 remaining studies had not been included in the training or validation datasets of the 2D or 3D algorithms and were automatically processed by the two algorithms.

## 2D/3D correlation

**Supplementary Figure S8. Scatter plot of LAVi_max_ of subjects with a 2D and 3D acquisition during the same examination.** The blue line depicts the simple linear regression, while the diagonal is shown for comparison (black dashed line). The coefficient of determination R^2^ of 0.75 provides a high positive correlation between the two calculated volumes.


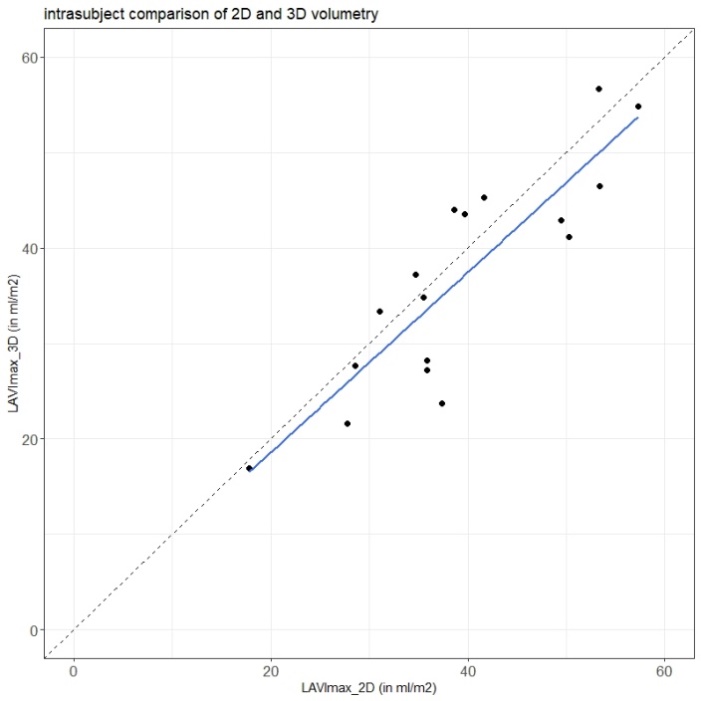

Supplement: Supplementary file 1 — Additional file 1. Contains descriptions of the studied populations, complementary results on segmentation and mitral valve classification, as well as detailed description of methods used for training and evaluation of the algorithms. Finally, a 3D cine segmentation algorithm is presented in detail. [file 12968_2021_791_MOESM1_ESM.docx]
